# Selective reduction of astrocyte apoE3 and apoE4 strongly reduces Aβ accumulation and plaque-related pathology in a mouse model of amyloidosis

**DOI:** 10.1186/s13024-022-00516-0

**Published:** 2022-02-02

**Authors:** Thomas E. Mahan, Chao Wang, Xin Bao, Ankit Choudhury, Jason D. Ulrich, David M. Holtzman

**Affiliations:** Department of Neurology, Hope Center for Neurological Disorders, Knight Alzheimer’s Disease Research Center, Washington University School of Medicine, 660 S. Euclid Ave, St. Louis, MO 63110 USA

**Keywords:** Alzheimer disease, Apolipoprotein E, apoE, Amyloid, Aβ, Aldh1l1-Cre, Astrocyte

## Abstract

**Background:**

One of the key pathological hallmarks of Alzheimer disease (AD) is the accumulation of the amyloid-β (Aβ) peptide into amyloid plaques. The apolipoprotein E (*APOE*) gene is the strongest genetic risk factor for late-onset AD and has been shown to influence the accumulation of Aβ in the brain in an isoform-dependent manner. ApoE can be produced by different cell types in the brain, with astrocytes being the largest producer of apoE, although reactive microglia also express high levels of apoE. While studies have shown that altering apoE levels in the brain can influence the development of Aβ plaque pathology, it is not fully known how apoE produced by specific cell types, such as astrocytes, contributes to amyloid pathology.

**Methods:**

We utilized *APOE* knock-in mice capable of having *APOE* selectively removed from astrocytes in a tamoxifen-inducible manner and crossed them with the APP/PS1-21 mouse model of amyloidosis. We analyzed the changes to Aβ plaque levels and assessed the impact on cellular responses to Aβ plaques when astrocytic *APOE* is removed.

**Results:**

Tamoxifen administration was capable of strongly reducing apoE levels in the brain by markedly reducing astrocyte apoE, while microglial apoE expression remained. Reduction of astrocytic apoE3 and apoE4 led to a large decrease in Aβ plaque deposition and less compact plaques. While overall Iba1^+^ microglia were unchanged in the cortex after reducing astrocyte apoE, the expression of the disease-associated microglial markers Clec7a and apoE were lower around amyloid plaques, indicating decreased microglial activation. Additionally, astrocyte GFAP levels are unchanged around amyloid plaques, but overall GFAP levels are reduced in the cortex of female apoE4 mice after a reduction in astrocytic apoE. Finally, while the amount of neuritic dystrophy around remaining individual plaques was increased with the removal of astrocytic apoE, the overall amount of cortical amyloid-associated neuritic dystrophy was significantly decreased.

**Conclusion:**

This study reveals an important role of astrocytic apoE3 and apoE4 on the deposition and accumulation of Aβ plaques as well as on certain Aβ-associated downstream effects.

**Supplementary Information:**

The online version contains supplementary material available at 10.1186/s13024-022-00516-0.

## Background

Alzheimer disease (AD) is the leading cause of dementia, affecting over 6 million Americans and ~ 50 million people worldwide [1, 2]. The strongest genetic risk factor for developing late-onset AD is apolipoprotein E (*APOE)* genotype. The influence of *APOE* on AD risk occurs in an isoform-dependent manner (ε2 < ε3 < ε4) [3–6]. One way apoE affects AD risk is through facilitating the formation of amyloid plaques, the earliest detectable pathological hallmark of AD [7–9]. ApoE is found as a constituent in amyloid plaques, suggesting it can directly facilitate plaque formation, and apoE affects amyloid deposition in an isoform and expression-level dependent manner [10–17]. *APOE4* carriers exhibit more amyloid pathology than non-carriers and mouse models of β-amyloidosis with the human *APOE4* gene knocked in develop more amyloid than those with *APOE3* [12, 15, 18–23]*.* The effect of apoE on Aβ is also dependent on the amount of apoE in the brain. Knockout of *Apoe* strongly reduces amyloid deposition in mouse models of amyloid deposition [13, 14, 24]. Mice that are hemizygous for *APOE* develop less Aβ plaque than mice homozygous for *APOE* [14, 15, 21, 25]. Additionally, reduction of apoE levels prior to plaque onset using apoE-targeted anti-sense oligonucleotides (ASO’s), results in reduced Aβ plaque pathology [26]. Conversely, overexpression of ApoE4 during the nascent stages of plaque formation leads to an increase in amyloid plaque deposition [27].

Within the brain, *APOE* is predominantly expressed by astrocytes under physiological conditions. However, when damage occurs in the brain, microglia significantly upregulate *APOE* expression [28–30]. In mouse models of amyloid pathology, plaque-associated microglia exhibit high levels of apoE expression as part of a broader “microglial neurodegenerative phenotype” (MGnD) or “disease-associated microglia” (DAM) transcriptional profile [28, 29, 31]. Microglial apoE expression may be critical for the microglial responses to injury in the brain since microglial activation is attenuated by germ line *Apoe* KO in mouse models of amyloid or tau pathology [24, 32]. Previous research from our lab has shown that microglia expressing human *APOE2, APOE3*, or *APOE4* produce apoE-containing lipoprotein particles that are smaller in size than particles produced by astrocytes [33]. The difference in apoE particle size in astrocytes and microglia raises the question as to whether or not apoE-containing lipoprotein particles produced by each cell type may have differential effects on the development of Aβ pathology.

Microglia-derived apoE can deposit within amyloid plaques and may contribute to plaque formation and influence morphology [34]. Other studies found selective removal of murine apoE from astrocytes in APPPS1ΔE9 mice reduces Aβ plaque burden [35]. Conversely, overexpression of *APOE4*, but not *APOE3*, in astrocytes exacerbated Aβ pathology, suggesting that astrocyte-derived human apoE may differentially affect amyloid pathology [27]. Whether astrocyte-specific expression of endogenously produced human *APOE* isoforms influences Aβ pathology, glial reactivity to Aβ plaque deposition, or downstream effects of Aβ deposition has not been investigated. To assess how the loss of astrocytic *APOE* impacts Aβ pathology, we used Aldh1l1-Cre/ERT2 BAC transgenic mice, in which the Aldh1l1 promoter drives expression of a tamoxifen-inducible Cre recombinase specifically in astrocytes, crossed with APPPS1-21/APOE3 or APOE4 knock-in mice.

## Methods

### Contact for reagent and resource sharing

Further information and requests for resources and reagents should be directed to David M. Holtzman (holtzman@wustl.edu).

### Experimental model and subject details

APPPS1–21 mice on a C57BL/6 N background (gift from Dr. Mathias Jucker, Department of Cellular Neurology, Hertie Institute for Clinical Brain Research, University of Tübingen, Germany) overexpress human APP bearing both the Swedish mutation and PSEN1 containing an L166P mutation, both driven by the Thy1 promoter [36]. ApoE3^flox/flox^ and apoE4^flox/flox^ (FE3 and FE4, respectively), human *APOE* knock-in mice on a C57BL background, were generated by replacing the mouse genomic sequence from the translation initiation codon in exon 2 to the termination codon in exon 4 with its human counterparts flanked by loxP site, driven by the endogenous *APOE* promoter [33]. Aldh1l1-Cre/ERT2 mice on a C57BL background were obtained from Jackson Laboratories (Stock No. 031008). To generate APPPS1-21/ apoE3^flox/flox^ or apoE4^flox/flox^ mice (APPPS1;FE3Cre- or APPPS1;FE4Cre-, respectively, and collectively referred to as Cre-), we crossed APPPS1-21 transgenic mice with FE3 or FE4 for several generations. To generate Aldh1l1-Cre/ERT2/apoE3^flox/flox^ or apoE4^flox/flox^ mice (AFE3 or AFE4, respectively), we crossed Aldh1l1-Cre/ERT2 mice to FE3 or FE4 for several generations. We then crossed APPPS1;FE3Cre- or APPPS1;FE4Cre- mice to AFE3 or AFE4 mice, respectively, to produce APPPS1-21/Aldh1l1-Cre/apoE3^flox/flox^ or apoE4^flox/flox^ mice (APPPS1;FE3Cre + or APPPS1;FE4Cre+, respectively, and collectively referred to as Cre+). Finally, we crossed APPPS1;FE3Cre + or APPPS1;FE4Cre + mice to FE3 or FE4 mice, respectively, to produce experimental mice utilized. All the experimental mice involved in the final analysis were obtained from the same generation. The sex of animals in each specific experiment can be found in the corresponding figure legends. All animal procedures and experiments were performed under guidelines approved by the Institutional Animal Care and Use Committee (IACUC) at Washington University School of Medicine. All of the phenotyping and data analysis was performed by researchers who were blind to the genotype of the mice.

## Methods details

### Tamoxifen administration

Tamoxifen was dissolved in corn oil at a concentration of 20 mg/ml by shaking overnight at 37 °C. After preparation, the tamoxifen solution was wrapped by foil, and stored at 4 °C for up to a month. Tamoxifen was given at 100 mg tamoxifen/kg body weight and administered via intraperitoneal (IP) injection once every 24 h for 6 consecutive days. All experimental mice received the same series of IP tamoxifen injections over 6 days.

### Brain isolation and preparation

Mice were anesthetized with 200 mg/kg pentobarbital and subsequently perfused with cold PBS containing 3 IU/ml heparin. After brain isolation, the left hemisphere was fixed in 4% paraformaldehyde for at least 24 h and then transferred to 30% sucrose and stored at 4 °C until sectioning. The right hemisphere was dissected into various parts (posterior- and anterior-cortex, hippocampus, etc.), all of which were snap-frozen using dry ice and stored at − 80 °C until further analysis.

### Histology and image acquisition

**Fig. 1:**
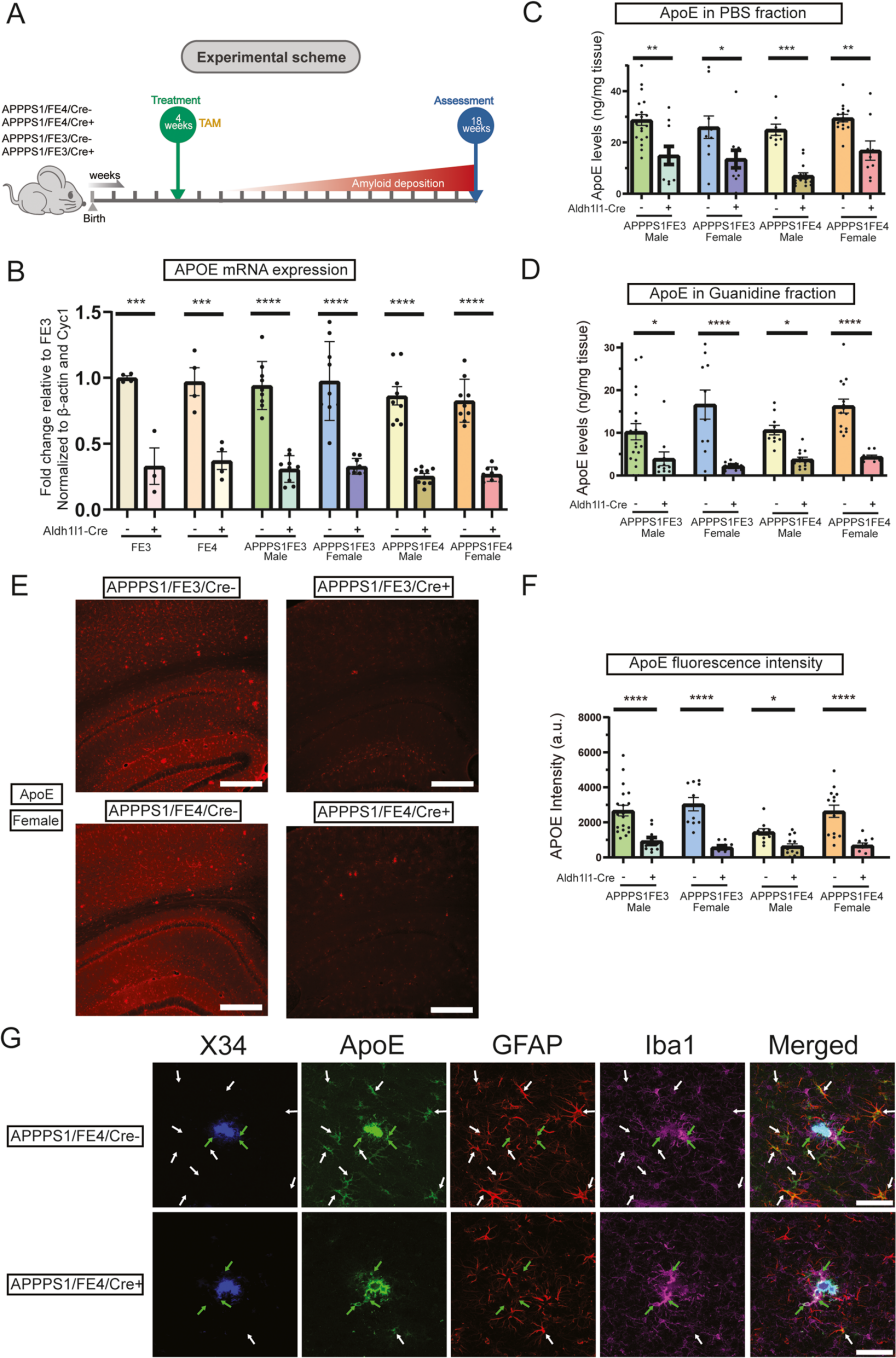
Tamoxifen administration reduces ApoE levels in Aldh1l1-Cre + APPPS1;FE3Cre- and APPPS1;FE4Cre- mice. **A** Timeline of the experimental scheme. Mice were given once-daily IP injections of tamoxifen (TAM) (100 mg TAM/kg body weight) at 4 weeks of age for 6 consecutive days. Sample collection and analysis occurred at 18 weeks of age. **B** *APOE* mRNA expression levels in Cre- and Cre + FE3, FE4, APPPS1;FE3, and APPPS1;FE4 mice. Cortical tissue samples were analyzed by qPCR (*n* = 9). **C** Soluble apoE levels in the cortex of Cre- or Cre + mice. Cortical tissue samples were homogenized in PBS and PBS-soluble apoE protein levels were analyzed by ELISA (*n* = 9–19). **D** Insoluble apoE levels in the cortex of Cre- or Cre + mice. PBS-insoluble cortex tissue samples from (C) were further homogenized in 5 M guanidine HCl to determine the amount of PBS-insoluble apoE that was guanidine-soluble. Protein levels were analyzed by ELISA (*n* = 9–19). **E** ApoE immunostaining in the cortex and hippocampus of Cre-and Cre + mice. Representative images are of female brain sections stained with an anti-apoE antibody. Scale bars = 300 μm. **F** Intensity of fluorescent apoE staining in Cre- or Cre + mice. The average pixel intensity was analyzed from images of apoE immunostained brain sections (*n* = 10–19). **G** Brain sections from female APPPS1;FE4Cre- and APPPS1;FE4Cre + mice co-stained for X-34 (blue), apoE (green), GFAP (red), and Iba1 (magenta). White arrows indicate co-localization of apoE with GFAP and green arrows indicate co-localization of apoE with Iba1. Scale bars = 50 μm. **A-G** * *p* ≤ 0.05, ** *p* ≤ 0.01, and **** *p* ≤ 0.0001; two-way ANOVA and Sidak’s post hoc test in (**B**), three-way ANOVA and Sidak’s post hoc test in (**D**); three-way ANOVA and uncorrected Fisher’s LSD test in (**C**) and (**F**). Data are expressed as mean ± SEM. See Supplementary Table 1 for detailed statistics

**Fig. 2:**
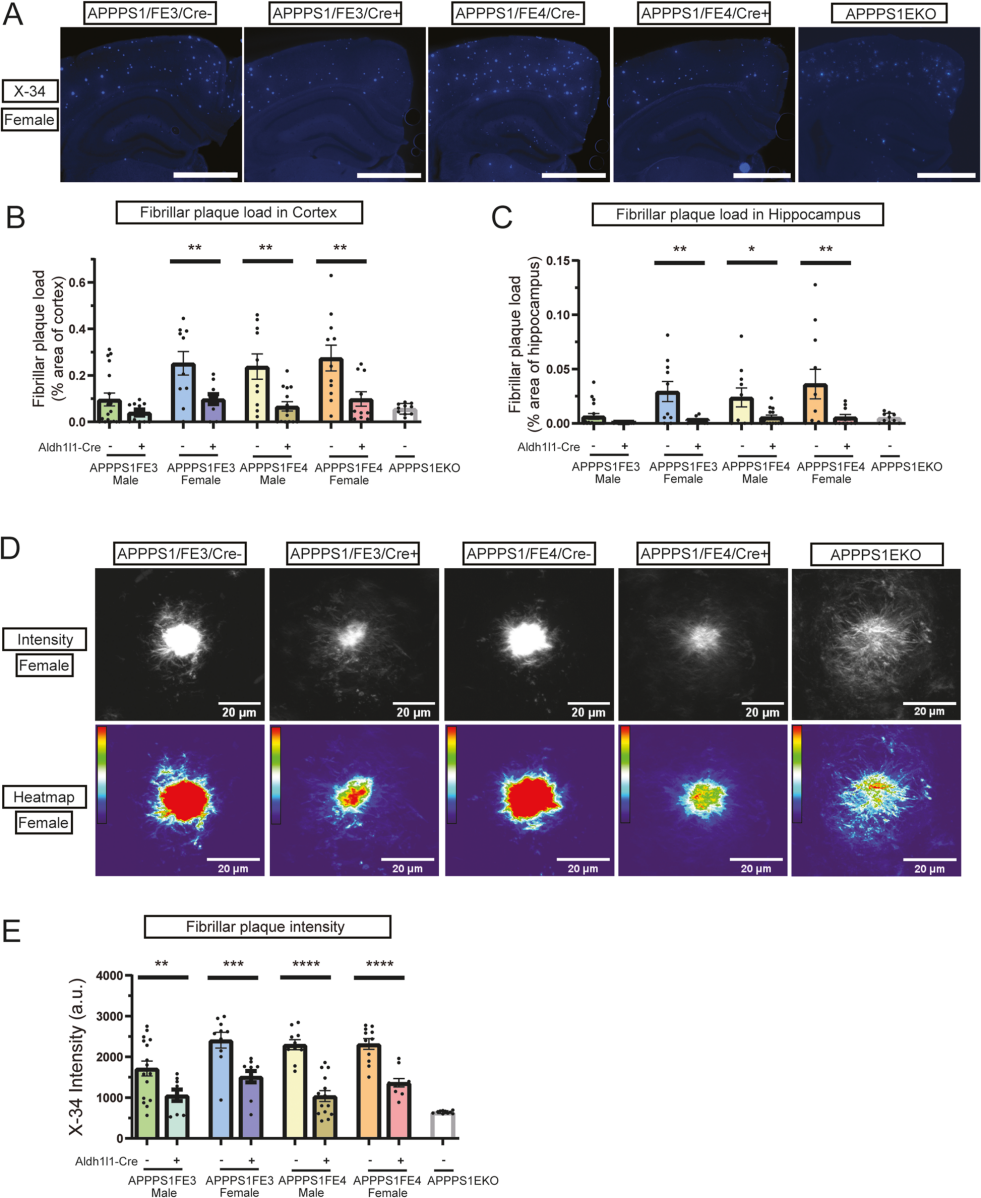
Reducing astrocytic apoE decreases fibrillar plaque levels and plaque intensity. **A** Fibrillar amyloid plaque staining in the cortex and hippocampus of female Cre-, Cre+, and APPPS1EKO mice. Representative images are of X-34 (blue) stained female brain sections. Scale bars = 1000 μm (**B**) Fibrillar plaque load in the cortex of Cre-, Cre+, and APPPS1EKO mice. Percent of cortex area covered by fibrillar plaque was determined by analyzing X-34 stained brain sections (*n* = 10–18). **C** Fibrillar plaque load in hippocampus of Cre-, Cre+, and APPPS1EKO mice. Percent of hippocampus area covered by fibrillar plaque was determined by analyzing X-34 stained brain sections (*n* = 10–18). **D** Intensity of fibrillar amyloid plaques in Cre-, Cre+, and APPPS1EKO mice. Representative images are of female X-34 stained amyloid plaques. Scale bars = 20 μm. **E** Measure of average pixel intensity of X-34 stained fibrillar plaques in the cortex of Cre-, Cre+, and APPPS1EKO mice (*n* = 9–16). **A-E** * *p* ≤ 0.05, ** *p* ≤ 0.01, *** *p* ≤ 0.001, and **** *p* ≤ 0.0001; three-way ANOVA and uncorrected Fisher’s LSD test in (**B**), (**C**), and (**E**). Data are expressed as mean ± SEM. See Supplementary Table 1 for detailed statistics

**Fig. 3:**
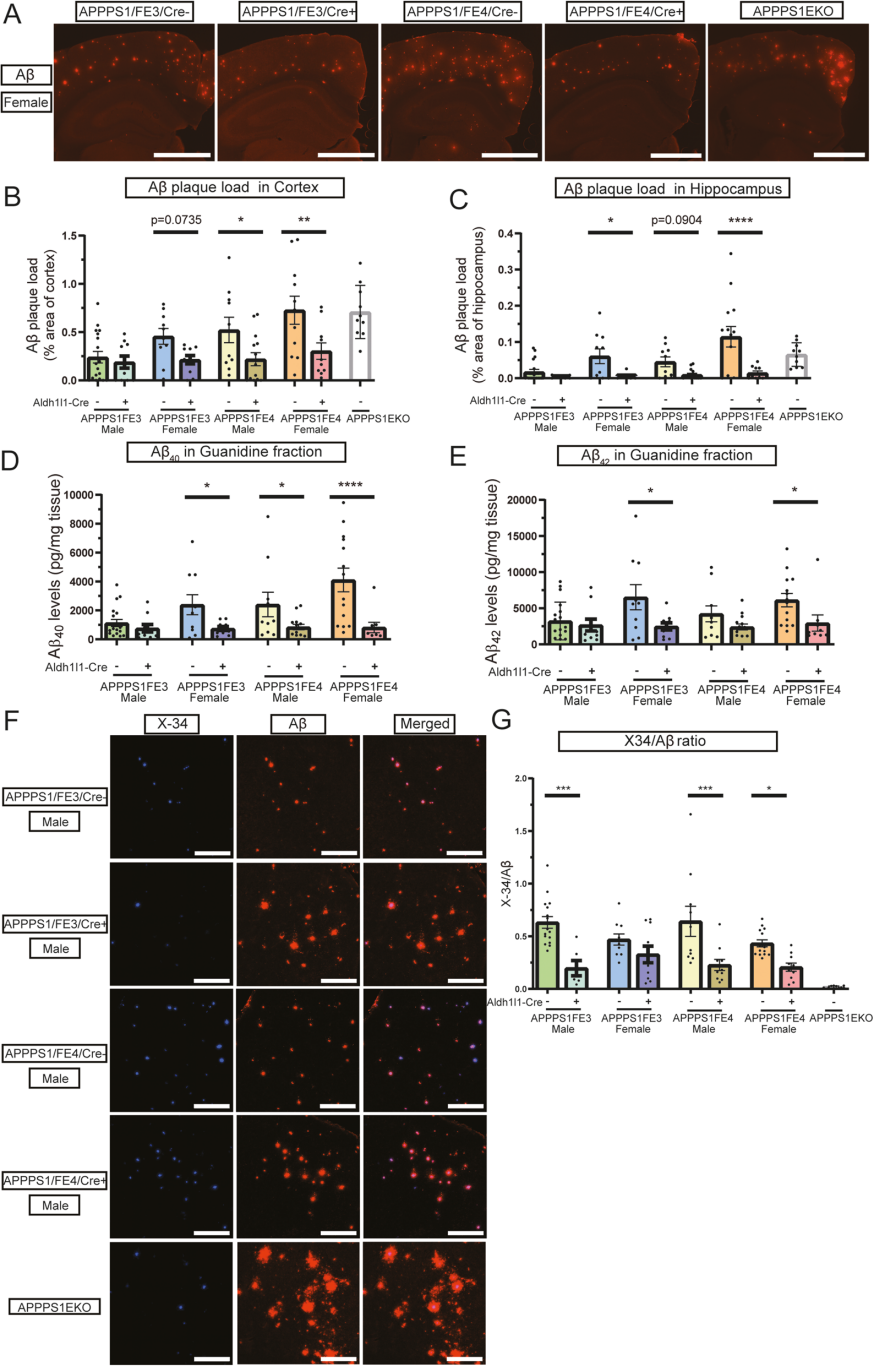
Reducing astrocytic apoE decreases Aβ plaque levels and alters Aβ deposition. **A** Aβ plaque staining in the cortex and hippocampus of Cre-, Cre+, and APPPS1EKO mice. Representative images are of Aβ immunostained female brain sections using the HJ3.4 anti-Aβ antibody (orange). Scale bars = 1000 μm. **B** Aβ plaque load in cortex of Cre-, Cre+, and APPPS1EKO mice. Percent of cortex area covered by Aβ plaque was determined by analyzing HJ3.4 stained brain sections (*n* = 10–18). **C** Aβ plaque load in the hippocampus of Cre-, Cre+, and APPPS1EKO mice. Percent of hippocampus area covered by Aβ plaque was determined by analyzing HJ3.4 stained brain sections (*n* = 10–18). **D** Insoluble Aβ_40_ levels in the cortex of Cre- and Cre + mice. PBS-insoluble cortical tissue samples that were further homogenized in 5 M guanidine HCl were analyzed by ELISA to determine the guanidine-soluble Aβ_40_ levels (*n* = 9–19). **E** Insoluble Aβ_42_ levels in the cortex of Cre- and Cre + mice. PBS-insoluble cortex tissue samples that were further homogenized in 5 M guanidine HCl were analyzed by ELISA to determine the guanidine-soluble Aβ_42_ levels (*n* = 9–19). **F** Deposition pattern of Aβ plaque and fibrillar amyloid plaque staining in Cre-, Cre+, and APPPS1EKO mice. Representative images are of X-34 (blue) and HJ3.4 (orange) co-stained male brain sections. Scale bars = 50 μm. **G** Ratio of fibrillar Aβ plaques to total Aβ deposition in Cre-, Cre+, and APPPS1EKO. The ratio was determined by dividing the area of X-34 staining by the area of HJ3.4 staining. (*n* = 6–16) (**A-F**) * *p* ≤ 0.05, ** *p* ≤ 0.01, *** *p* ≤ 0.001, and **** *p* ≤ 0.0001; three-way ANOVA and Sidak’s post hoc test in (**B**) and (**C**); three-way ANOVA and uncorrected Fisher’s LSD test in (**D**), (**E**), and (**G**). Data are expressed as mean ± SEM. See Supplementary Table 1 for detailed statistics

**Fig. 4:**
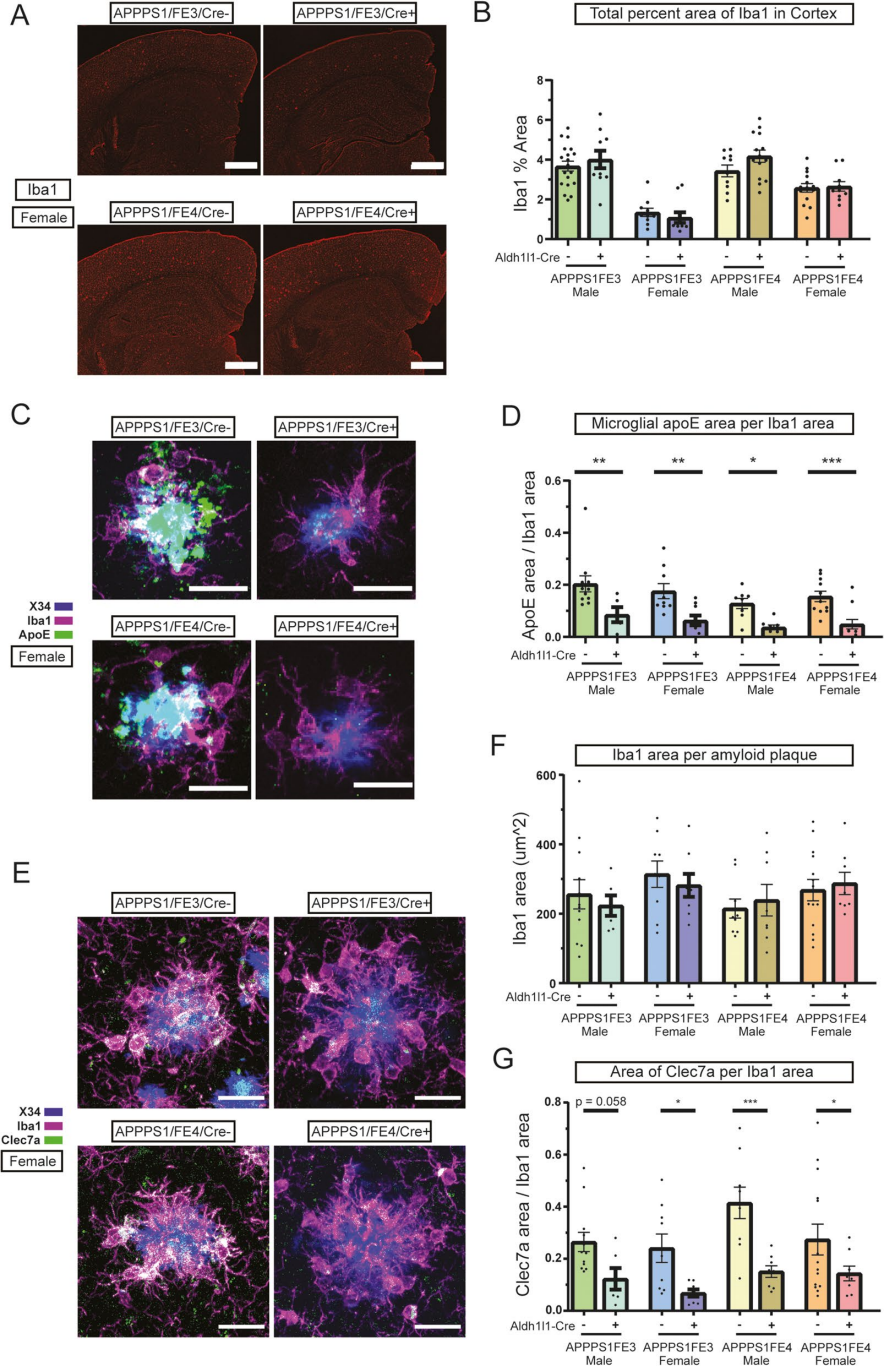
Microglial activation is reduced in mice with a decrease in astrocytic apoE. **A** Microglia staining in the cortex and hippocampus of female Cre- and Cre + mice. Representative images are of female brain sections immunostained using an anti-Iba1 antibody (red). Scale bars = 200 μm. **B** Microglial coverage in the cortex of Cre- and Cre + mice. Percent of cortex area covered by microglia was determined by analyzing Iba1 stained brain sections (*n* = 10–19). **C** ApoE+ microglia around fibrillar amyloid plaques in female Cre- and Cre + mice. Representative images are of apoE immunostaining (green) using an anti-apoE antibody and microglia immunostaining using an anti-Iba1 antibody (magenta) around X-34 stained (blue) amyloid plaques. ApoE co-localized with Iba1 appears white. Scale bars = 20 μm. **D** Microglial apoE levels around fibrillar amyloid plaques in Cre- and Cre + mice. Area of apoE+ microglia per total microglia area was determined by analyzing the level of apoE+ microglia staining to total microglia staining within 10 μm of X-34 stained plaques (*n* = 5–12). **E** Microglia clustering and activated microglia around fibrillar amyloid plaques in female Cre- and Cre + mice. Representative images are of Clec7a immunostaining (green), using an anti-Clec7a antibody, and microglia immunostaining using an anti-Iba1 antibody (magenta) around X-34 stained (blue) amyloid plaques. Clec7a co-localized with Iba1 appears white. Scale bars = 20 μm. **F** Clustering of microglia around fibrillar amyloid plaques in Cre- or Cre + mice. Area covered by microglia around fibrillar amyloid plaques was determined by analyzing the level of Iba1 staining within 15 μm of X-34 stained plaques (*n* = 6–14). **G** Coverage of activated microglia around fibrillar amyloid plaques in Cre- or Cre + mice. Area of activated microglia around fibrillar amyloid plaques was determined by analyzing the level of cleca7a staining within 15 μm of X-34 stained plaques per total area of Iba1 (*n* = 6–14). **A-G** * *p* ≤ 0.05, ** *p* ≤ 0.01, *** *p* ≤ 0.001; three-way ANOVA and uncorrected Fisher’s LSD test in (**B**), (**D**), (**F**), and (**G**). Data are expressed as mean ± SEM. See Supplementary Table 1 for detailed statistics

**Fig. 5:**
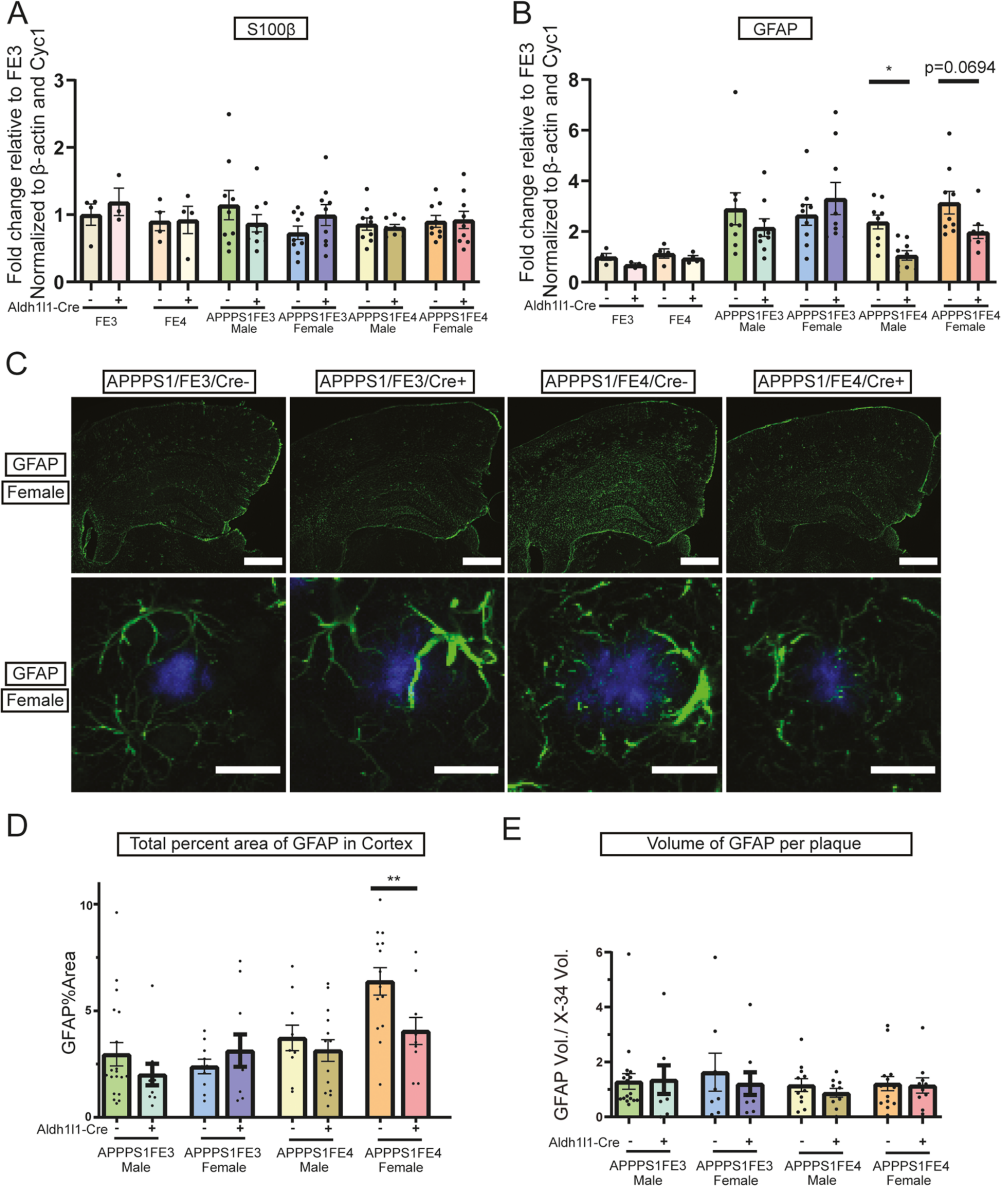
Astrocyte activation is reduced in APPPS1;FE4Cre + mice. **A** Gene expression analysis of S100β in Cre-, Cre + FE3Cre-, FE3Cre+, FE4Cre-, and FE4Cre + mice. Graph is of S100β gene levels assessed by qPCR from cortical tissue samples (*n* = 3–9). **B** Gene expression analysis of GFAP in Cre-, Cre + FE3Cre-, FE3Cre+, FE4Cre-, and FE4Cre + mice. Graph is of GFAP gene levels assessed by qPCR from cortical tissue samples (*n* = 3–9). **C** Activated astrocyte staining in female Cre- and Cre + mice. Representative images in the top panel are of the cortex and hippocampus from brain sections immunostained using an anti-GFAP antibody (green). Images in the bottom panels are of GFAP immunostaining (green), using an anti-GFAP antibody, around X-34 stained amyloid plaques (blue). Scale bars = 200 μm (top panels), 20um (bottom panels). **D** Astrocyte activation in the cortex of Cre- or Cre + mice. Percent of cortical area covered by activated astrocytes was determined by analyzing GFAP stained brain sections (*n* = 10–14). **E** Astrocyte activation around fibrillar amyloid plaques in Cre- and Cre + mice. The volume of activated astrocyte processes around fibrillar amyloid plaques was determined by analyzing the amount of GFAP staining within 15 μm of X-34 stained plaques. GFAP volume was divided by the X-34 volume to normalize to the amount of plaque and account for differences in plaque size (*n* = 8–19). **A-D** * *p* ≤ 0.05, ** *p* ≤ 0.01; three way ANOVA and uncorrected Fisher’s LSD test in (**A**), (**B**), (**D**), and (**E**). Data are expressed as mean ± SEM. See Supplementary Table 1 for detailed statistics

**Fig. 6:**
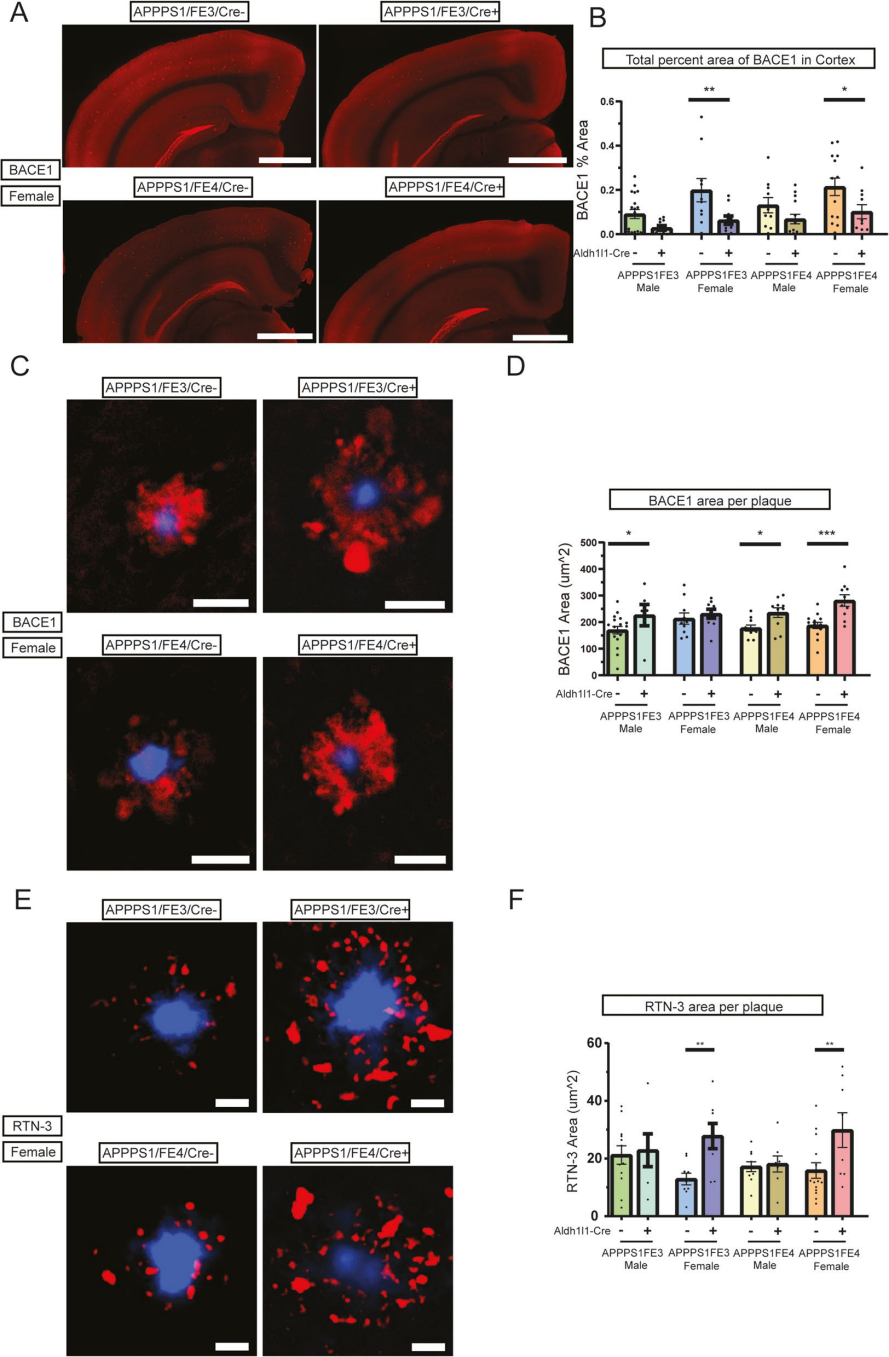
Neuritic dystrophy is increased around plaques, but decreased overall, with a reduction in astrocytic apoE. **A** Dystrophic neurite staining in the cortex and hippocampus of Cre- and Cre + mice. Representative images are of brain sections immunostained using an anti-BACE1 antibody (red). Scale bars = 1000 μm. **B** Level of neuritic dystrophy in the cortex of Cre- and Cre + mice. Percent of cortical area covered by dystrophic neurites was determined by analyzing BACE1 stained brain sections (*n* = 10–19). **C** Dystrophic neurites around fibrillar amyloid plaques in Cre- and Cre + mice (BACE1). Representative images are of BACE1 immunostaining (red), using an anti-BACE1 antibody, around X-34 stained (blue) amyloid plaques. Scale bars = 20 μm. **D** Level of neuritic dystrophy around fibrillar amyloid plaques in Cre- and Cre + mice (BACE1). Percent of area covered by dystrophic neurites around fibrillar amyloid plaques was determined by analyzing the level of BACE1 staining within 15 μm of X-34 stained plaques (*n* = 6–18). **E** Dystrophic neurites around fibrillar amyloid plaques in Cre- and Cre + mice (RTN-3). Representative images are of female RTN-3 immunostaining (red), using an anti-RTN-3 antibody, around X-34 stained (blue) amyloid plaques. Scale bars = 20 μm. **F** Level of neuritic dystrophy around fibrillar amyloid plaques in Cre- and Cre + mice (RTN-3). Percent of area covered by dystrophic neurites around fibrillar amyloid plaques was determined by analyzing the level of RTN-3 staining within 15 μm of X-34 stained plaques (*n* = 6–14). **A-D** * *p* ≤ 0.05, ** *p* ≤ 0.01, and **** *p* ≤ 0.0001; three-way ANOVA and Sidak’s post hoc test in (**B**); three-way ANOVA and uncorrected Fisher’s LSD test in (**D**) and (**F**). Data are expressed as mean ± SEM. See Supplementary Table 1 for detailed statistics

Hippocampal-containing sections were selected for human apoE (Cell Signaling, 13,366, 1:500), GFAP (Abcam, ab53554, 1:500; Millipore, MAB3402B, 1:2000), Iba1 (Abcam, ab5076, 1:500; Wako, 019–19,741, 1:5000), Aβ (HJ3.4, in house, mouse monoclonal, 2 μg/ml), Clec7a (InviviGen, mabg-mdect, 1:50), BACE1 (Abcam, ab108394, 1:100), and RTN-3 (a generous gift from Dr. Riqiang Yang, 1:1000) immunofluorescence staining. For reticulon-3 (RTN-3) staining, sections were pre-mounted on to Superfrost + glass microscope slides. For all other stainings, sections were free-floating. Sections were washed in Tris-buffered saline (TBS) buffer for 3 times, 5 min each. After washing, sections were incubated in TBS with 0.25% Triton X-100 (TBSX) for 30 min at room temperature to permeabilize the sections, followed by 1 time TBS washing for 5 min. Then sections were incubated with 2 μM X34 in the X-34 staining buffer (40% ethanol, 60% TBS, 1:500 vol. 10 N NaOH) for 20 min. After X-34 staining, sections were washed 3 times for 2 min each in the X-34 washing buffer (40% ethanol and 60% TBS), followed by 3 times washing in TBS for 5 min each. For RTN-3 staining, antigen retrieval was performed by heating sections to 95 °C in 50 nM Sodium Citrate buffer for 30 min followed by 3 times washing in TBS for 5 min each. After washing, sections were blocked by 10% donkey serum in TBSX for 1 h at room temperature to prevent non-specific binding. Then sections were incubated with primary antibodies at 4 °C, overnight. After overnight incubation, sections were washed 3 times in TBS for 5 min each. Then sections were incubated with corresponding secondary fluorescence antibodies (Life Technologies) for 2 h at room temperature. After 3 times washing by TBS for 10 min each, sections were mounted to glass slides. Slides were coverslipped by ProLong Glass Antifade Mountant (Invitrogen, P36980) and scanned by Nikon A1Rsi Confocal Microscope, Leica Stellaris 5, or BioTek Cytation5. Representative images in Fig. 1E were captured by the BioTek Cytation5 using a 10X objective. Representative images in Figs. 2A, 3A, 4A, 5C (top panel), 6A, S1, and S2A were captured by the BioTek Cytation5 using a 4X objective. Representative images in Figs. 3F, 6C, E, S2B, and S5 were captured by the BioTek Cytation5 using a 20X objective. Representative images in Figs. 1G, 2D, 4C, and E were captured by the Leica Stellaris 5 using a 40X oil objective. Representative images in Fig. 5B (bottom panel) were captured by the Nikon A1Rsi using a 40X objective.

### Image processing and quantification

Acquired images were analyzed by using Image J v1.53c (https://imagej.net/Fiji), Imaris 9 (https://imaris.oxinst.com/), and BioTek Gen5 (https://www.biotek.com/products/software-robotics/) software. ApoE fluorescent staining intensity in Fig. 1F was determined using images captured by the BioTek Cytation5. The average pixel intensity for each image was found by setting a minimal threshold to highlight positive staining, running the “Analyze Particle” function to obtain the mean pixel intensity, and then subtracting the average background pixel intensity. The average background pixel intensity was determined by calculating the average pixel intensity of APPPS1EKO images. To determine the percent of hippocampal area or cortical area covered by X-34, HJ3.4, Iba1, GFAP, or BACE1 in Figs. 2B, C, 3B, C, 4B, 5D, and 6B, images were analyzed as previously described [37, 38]. Briefly, Image J software was used to analyze images captured with the BioTek Cytation5. Regions of interest (ROI’s) of images were traced, images were thresholded to highlight positive staining, and the “Analyze Particle” function was used to obtain the percent area covered. While intense BACE1 staining was present in the hippocampus, quantification of the hippocampus was excluded because high levels of BACE1 in the mossy fibers are physiologically normal and not indicative of any neuritic dystrophy. The average intensity of fibrillar plaques in Fig. 2E was also found using ImageJ software to analyze images of X-34 stained sections captured with the BioTek Cytation5 software. The cortex was traced, images were thresholded to highlight positive staining, and the “Analyze Particle” function was used to obtain the average pixel intensity. The X-34 to HJ3.4 ratio in 3G was determined using images of the cortex captured with the BioTek Cytation5 and analyzed using ImageJ software. Images were thresholded to highlight positive staining, and the “Analyze Particle” function was used to obtain the percent area covered. The area of X-34 was then divided by the area of HJ3.4. To determine the percent area of Clec7a and Iba1 staining around X-34 plaques in Fig. 4E, ImageJ software was used to analyze images captured with the Leica Stellaris 5 confocal microscope. The images of X-34^+^ plaques were thresholded to highlight plaques, “Analyze Particles” was run, and the thresholded plaque ROI’s were combined and then enlarged by 15 μm. The enlarged ROI’s were then transferred to the corresponding Clec7a and Iba1 images, images were thresholded, and “Analyze Particles” was run to find the area of Clec7a and Iba1. The area of Clec7a was then divided by the area of Iba1. The GFAP volume to X-34 volume in Fig. 5D was found using Imaris software to analyze images obtained by the Nikon A1Rsi confocal microscope, as previously described [24]. Briefly, Surfaces were created for X-34 and GFAP and the Dilate Xtension was used to dilate the X-34 surfaces by 15 μm. The surface-surface co-localization Xtension was run using the dilated X-34 surfaces and GFAP surfaces and the volume of GFAP within 15 μm of X-34 plaque was determined based on overall X-34 plaque volume. The BACE1 and RTN-3 area per X-34 plaque was determined using BioTek Gen5 software to analyze images obtained by the BioTek Cytation5. The Cellular Analysis function was used with the primary mask thresholded and set based on the X-34 staining. The secondary mask was set using a 15 μm expanded distance from the X-34 primary mask, BACE1 and RTN-3 staining was thresholded, and the average BACE1 area per X-34 plaque and RTN-3 area per X-34 plaque was determined. To determine the amount of apoE area per Iba1 area around plaques, images were obtained with the Leica Stellaris5 confocal microscope. The images of X-34^+^ plaques were subtracted from the corresponding apoE images to remove any apoE signal co-localized with X-34, leaving only non-plaque associated apoE. The images of X-34^+^ plaques were then thresholded to highlight plaques, “Analyze Particles” was run, and the thresholded plaque ROI’s were combined and then enlarged by 10 μm. The enlarged ROI’s were then transferred to the corresponding apoE and Iba1 images, images were thresholded, and “Analyze Particles” was run to find the area of apoE and Iba1 around the plaque. To determine what apoE was located within microglia, the Iba1 images were subtracted from the corresponding apoE images to leave apoE not co-localized with Iba1. The remaining area of apoE around plaques that was not co-localized with Iba1 was determined using the enlarged ROI, thresholding the images, and running “Analyze Particles”. These areas were then subtracted from the total apoE area around plaques to determine the amount of apoE area that was co-localized with Iba1. The apoE area co-localized with Iba1 was then divided by the total area of Iba1 around the plaques.

### Brain tissue sample processing

Mouse posterior cortical tissue samples were sequentially homogenized with cold PBS, and then 5 M guanidine buffer in the presence of 1X Complete Protease Inhibitor (Roche, 11,697,498,001) and 1X phosSTOP phosphatase Inhibitor (Roche, 04906845001). First, tissues were weighed, a half spoon of beads (Next Advance, ZrOB05) were added, and samples were homogenized for 45 s on setting 3, using a bead homogenizer (Next Advance, Bullet Blender Strom 24), in cold PBS buffer at 20 μl buffer/1 mg tissue. Homogenates were centrifuged 30 min at 15,000 rpm at 4 °C. The supernatant was saved as the PBS soluble fraction. Then the same amount of 5 M guanidine buffer was added to the pellet and homogenized on bead homogenizer for 3 min on setting 8, followed by 1 h rotation at room temperature. Finally, homogenates were centrifuged for 30 min at 15,000 rpm at 4 °C. The supernatant was saved as the 5 M guanidine insoluble fraction. All fractions were stored at − 80 °C until further analyzed.

### Sandwich ELISA

The levels of apoE, Aβ_40_, and Aβ_42_ in PBS and 5 M guanidine fractions were measured by sandwich ELISA and normalized to the tissue weight. The coating antibodies for human apoE, Aβ_40_, and Aβ_42_ were HJ15.3 (in house, mouse monoclonal, 5 μg/ml), HJ2 (in house, mouse monoclonal, 20 μg/ml), and HJ7.4 (in house, mouse monoclonal, 10 μg/ml), respectively [33, 39]. The capture antibodies were HJ15.7-biotinylated (in house, mouse monoclonal, 150 ng/ml) for human apoE and HJ5.1-biotinylated (in house, mouse monoclonal, 90 ng/ml) for both Aβ_40_ and Aβ_42_ [33, 39].

### Fluidigm biomark HD real-time PCR

Both male and female mouse anterior cortex tissues were used for the gene expression analysis. Nine samples from all APPPS1 positive groups were selected based on the mean values of the X34 plaque load. Four samples from APPPS1 negative FE3Cre-, FE4Cre-, and FE4Cre + groups, 3 samples from APPPS1 negative FE3Cre + group were also used as the APPPS1 negative controls. mRNA was exacted from frozen tissues using RNeasy Micro Kit (Qiagen, 74,004) and converted to cDNA using the high-capacity RNA-to-cDNA kit (Applied Biosystems, 4,387,406), following the manufacturer’s instructions. Gene expression was conducted using Fluidigm Biomark HD Real-Time PCR System in collaboration with Genome Technology Access Core at Washington University. Using Taqman primers (Life Technologies), the fold-changes were relative to FE3Cre- after values were normalized to the mean values of β-actin and Cyc1.

### Western blotting

Mouse cortical tissues were lysed using RIPA buffer (4°C). Samples were randomly selected and included 3 males and 3 females in each group. Complete Protease Inhibitor and phosSTOP Phosphatase Inhibitor were added freshly to the RIPA buffer. After weighing tissues, 50 μl cold RIPA/1 mg tissue and 1 spoon of beads (ZrOB05) were added to the tissue-containing Safe-Lock microcentrifuge tubes. Tissues were homogenized using a bead homogenizer (Bullet Blender Strom 24) on setting 8 for 3 min. Homogenates were centrifuged in a microcentrifuge at 15,000 rpm for 30 min at 4°C. Supernatant was collected for western blot. Micro BCA Protein Assay Kit was utilized to measure protein concentration. Five micrograms of total protein was loaded and separated by 4–12% NuPAGE gels in MOPS buffer. Gel was transferred for 7 min on a nitrocellulose membrane using the iBlot 2 system. After washing 3 times for 5 min each with TBS buffer, including 0.05% Tween (TBST), the membrane was blocked with 5% milk in TBST for 1 h at room temperature, followed by incubation with APP C-terminal antibody (Sigma, rabbit polyclonal, A8717, 1:1000) and α-tubulin antibody (mouse monoclonal, 66,031–1-lg, 1:15,000) at 4 °C. After overnight incubation, the membrane was incubated with corresponding secondary HRP-conjugated secondary antibodies for 1 h at room temperature. Images were captured using the ChemiDoc™ MP Imaging System (BIO-RAD). Full length and C-terminal fragments of APP, as well as α-tubulin bands were captured separately and analyzed by ImageJ software.

### Quantification and statistical analysis

All values were reported as mean ± SEM. All statistical analyses were conducted in Prism 8 (GraphPad). Two-way or Three-way ANOVA was used for assessing significance between more than two groups. *P* values less than 0.05 (*p* < 0.05) were considered significant for all tests. *p < 0.05, **p < 0.01, ***p < 0.001, ****p < 0.0001. The significant *p* values and F values for each experiment can be found in Supplementary Table 1. The value of n per group and what n represents in each specific experiment can be found in the corresponding figure legends and in Supplementary Table 1.

## Results

### ApoE levels following tamoxifen administration

In order to assess how the loss of astrocytic apoE impacts Aβ pathology, we used Aldh1l1-Cre/ERT2 BAC transgenic mice to selectively remove *APOE* from astrocytes in a tamoxifen dependent manner. APPPS1;FE3Cre + and APPPS1;FE4Cre + mice (Cre+) were created to assess Aβ plaque pathology compared to APPPS1;FE3Cre- and APPPS1;FE4Cre- mice (Cre-). Once-daily intraperitoneal injections of tamoxifen were administered to mice in each group at 4 weeks of age (2–4 weeks before the initial formation of Aβ plaques in these mice) for 6 consecutive days (Fig. 1A). All mice received the same series of tamoxifen injections, including the Cre- mice that served as the control. A cohort of Cre + and Cre- mice were collected at 6 weeks of age, 1 week after completing tamoxifen administration, and showed no signs of Aβ pathology (data not shown). To investigate the efficiency of *APOE* removal from astrocytes after tamoxifen administration, we first assessed apoE mRNA and protein levels in the brain at 18-weeks of age (Fig. 1A). ApoE mRNA levels were assessed by qPCR and were significantly reduced by ~ 70% in the Cre + mice compared to the Cre- mice (Fig. 1B). To assess apoE protein levels, cortex tissue samples were sequentially extracted in PBS and guanidine buffers to measure soluble and insoluble apoE, respectively. Soluble apoE levels were also significantly reduced by ~ 50–70% in all Cre + mice compared to Cre- mice (Fig. 1C). Insoluble apoE levels, which mostly reflects apoE co-deposition in Aβ plaques [40, 41], were also significantly reduced in Cre + mice compared to Cre- mice, with female mice having a ~ 75–85% reduction and male mice having a ~ 60–65% reduction (Fig. 1D).

Next, we assessed the cell-type-specificity of apoE expression after tamoxifen administration by performing immunofluorescent staining of apoE (Fig. 1D). We observed a strong overall reduction in the intensity of apoE staining in the cortex and hippocampus by ~ 60–80% for the Cre + mice compared to the Cre- mice (Fig. 1E and F). Since apoE is secreted into the extracellular space in lipoprotein particles, the overall intensity of the apoE immunostaining in both the cortex and hippocampus may be influenced by a combination of both intracellular and extracellular apoE. Additionally, plaques are primarily located in the cortex at the ages we studied and thus the intense foci of apoE staining in the cortex is likely from apoE associated with amyloid deposits. Qualitative assessment of the staining for apoE also revealed a strong decrease in the presence of apoE in GFAP+ astrocytes. In the Cre- mice nearly all GFAP^+^ astrocytes were also apoE^+^ while the Cre + mice exhibited only rare apoE staining in GFAP^+^ astrocytes (Fig. 1G). We also observed apoE to still be present in Iba1^+^ microglia, particularly in the vicinity of Aβ plaques (Fig. 1G). Therefore, the tamoxifen administration was able to effectively reduce the overall apoE levels and strongly reduce apoE expression in astrocytes, while appearing to still enable microglial to express apoE in the Cre + mice. Additionally, while the immunostaining showed that overall apoE levels were greatly reduced in the Cre + mice, there were still intense foci of apoE staining co-localized with the X-34^+^ amyloid plaques, similar to what was observed in the Cre- mice (Fig. 1E and G).

### Aβ plaque accumulation in mice lacking astrocytic *APOE*

The deposition of Aβ into fibrillar amyloid plaques is influenced by the expression level and isoform of apoE. With the targeted removal of astrocytic *APOE* resulting in a significant reduction in overall apoE levels, we investigated what impact this reduction might have on the deposition of Aβ into X34^+^ fibrillar amyloid plaques (Fig. 2A, S1). In the cortex and hippocampus, there was a large and significant decrease in fibrillar plaque load in APPPS1;FE3Cre + females and in APPPS1;FE4Cre + males and females, while the male APPPS1;FE3Cre + mice did not show a significant difference (Fig. 2B and C). In the cortex, the female Cre + and APPPS1;FE4Cre + males had a ~ 60–70% reduction in amyloid burden and in the hippocampus the reduction was ~ 75–85%. Overall, the total amyloid burden with the loss of astrocytic apoE was qualitatively similar to that observed in APPPS1;EKO mice (Fig. 2B and C). An analysis of fibrillar plaque intensity revealed Cre + mice have a significantly reduced overall intensity compared to the Cre- mice (Fig. 2D and E) and exhibit a smaller, less dense compact core. The reduction in plaque intensity for the Cre + mice was ~ 40–55% compared to the intensity of the Cre- plaques. However, a qualitative assessment of the APPPS1;EKO mice showed their fibrillar plaques form with a core that is even less compact and less intense than the Cre + mice (Fig. 2D and E).

To assess overall Aβ deposition, staining was performed using an anti-Aβ antibody (Fig. 3A, S2A). In the cortex, male APPPS1;FE3Cre + mice showed no difference in Aβ levels, while female APPPS1;FE3Cre + mice showed a trend towards lower Aβ plaque load compared to female APPPS1;FE3Cre- mice (Fig. 3B). For the APPPS1;FE4Cre + mice, both male and female mice had a significant decrease in cortical plaque coverage of ~ 60% compared to APPPS1;FE4Cre- mice (Fig. 3B). In the hippocampus, the Cre + females had significantly lower Aβ plaque coverage with a ~ 90% reduction (Fig. 3C). Interestingly, a qualitative analysis of the APPPS1;EKO mice revealed a higher Aβ plaque load than the Cre + mice (Fig. 3B and C)*.*

To further analyze the accumulation of Aβ in the brain, the level of insoluble Aβ was assessed in the guanidine soluble (PBS-insoluble) fractions from homogenized cortical tissue samples. Both Aβ_40_ and Aβ_42_ levels from the guanidine fraction were assessed by ELISA. Aβ that is insoluble in PBS and detected in the guanidine fraction serves as a measure of how much Aβ has accumulated in deposits in the brain and constitutes the majority of the Aβ pool once Aβ aggregates. The Aβ_40_ and Aβ_42_ in the PBS soluble fraction showed no difference between each of the groups (data not shown). The APPPS1;FE3Cre + males showed no difference in Aβ_40_ and Aβ_42_ levels in the guanidine fraction compared to the APPPS1;FE3Cre- male mice while Aβ_40_ was significantly decreased in the APPPS1;FE4Cre + males by 65%. Both the Aβ_40_ and Aβ_42_ levels were significantly decreased by in the Cre + females compared to the Cre- females, with a ~ 70–80% decrease for Aβ_40_ and a ~ 50–60% decrease for Aβ_42_ (Fig. 3D and E).

An assessment of the Aβ immunostaining in relation to the formation of fibrillar Aβ plaques also revealed unique patterns of Aβ deposition in the Cre + mice compared to the Cre- mice (Fig. 3F, S2B). In the Cre + mice, the fibrillar Aβ core was smaller with a greater percent of each plaque consisting of non-fibrillar Aβ (Fig. 3F). However, the APPPS1;EKO mice had an even greater and more dispersed accumulation of non-fibrillar Aβ than the APPPS1;FE3Cre + and APPPS1;FE4Cre + mice (Fig. 3F). In order to assess how much of the total Aβ that was accumulating was forming fibrillar amyloid structures, the amount of fibrillar Aβ detected by X-34 staining was compared to the total Aβ stained by HJ3.4. Immunostaining with HJ3.4 detects all forms of Aβ, so all X-34 positive Aβ plaques will also be detected by HJ3.4 staining, but not all Aβ detected with HJ3.4 will be positive for X-34. Therefore, comparing the amount of X-34 staining to the total amount of HJ3.4 staining provides insight into differences in the structural formation of plaques. The ratio of the X-34 area to HJ3.4 area revealed that the male Cre + mice had an approximate 65% reduction in the X-34 to HJ3.4 ratio, while for the females only the APPPS1;FE4Cre + mice had a significant reduction in the ratio of about 50% (Fig. 3G).

Overall, the biochemical results, combined with the Aβ staining results, show that the loss of astrocytic human apoE leads to a decrease in overall Aβ pathology compared to mice with no loss of apoE. Additionally, the loss of astrocytic apoE leads to more diffuse, less fibrillar plaques, though not as diffuse as is seen with a complete knockout of apoE. The loss of astrocytic apoE also reduces the total amount of fibrillar amyloid plaques that form in relation to the total amount of Aβ that deposits in the parenchyma. However, the changes observed in the overall Aβ plaque load, and in the types of Aβ plaques that form, could result from changes in the overall APP levels and/or in the processing of APP that occur with the loss of astrocytic apoE. To address this concern, we performed Western blotting of tissue samples for full-length APP and APP-CTFs and saw no differences between groups (Fig. S2C, S2D), indicating there were no differences in APP levels or in the processing APP after removal of astrocytic apoE.

### Microglial activation after the loss of astrocytic apoE

The activation state of microglia has been shown to be influenced by apoE isoform and the presence of Aβ plaque pathology in the brain. To better understand how the loss of astrocytic apoE was influencing microglial gene expression, we performed RT-qPCR on cortical tissue samples. The APPPS1;FE3Cre- and APPPS1;FE4Cre- mice exhibited an upregulation of DAM-associated transcripts compared to FE3 or FE4 mice, respectively, indicative of a microglial response to amyloid plaques (Fig. S3D-J). The APPPS1;FE3Cre + and APPPS1;FE4Cre + mice also showed some upregulation of DAM associated genes, however the increase in these Cre + mice was reduced in comparison to the Cre- mice for several of the DAM genes (Fig. S3D-I), including *Clec7a* (Fig. S3F).

To assess if the overall levels of microglia were changed in the Cre + mice, potentially accounting for differences in overall DAM gene expression, brain sections were stained for Iba1 (Fig. 4A). Overall, the % area of the cortex covered by Iba1 staining remained unchanged between the APPPS1;FE3Cre + and APPPS1;FE3Cre- mice and between the APPPS1;FE4Cre + and APPPS1;FE4Cre- mice (Fig. 4B). However, the apoE3 female mice did show lower overall Iba1 staining levels than the other groups. To investigate the state of microglia surrounding Aβ plaques, an analysis of the amount of Clec7a, a marker of DAM microglia, was assessed based upon the total amount of Iba1+ microglia present (Fig. 4E). The amount of Iba1+ microglia clustering around X-34 plaques was unchanged between the Cre- and Cre + groups, indicating the loss of astrocytic apoE did not alter the ability of microglia to migrate to the plaques (Fig. 4F). However the level of Clec7a was shown to be decreased by ~ 50–70% around fibrillar plaques in the Cre + mice compared to the Cre- mice, (Fig. 4G). Furthermore, an assessment of the presence of apoE (which is also a marker of DAM microglia) in plaque associated microglia also showed a significant reduction of ~ 60–70% in Cre + mice compared to Cre- mice (Fig. 4C, D) The reduction of both microglial apoE and Clec7a levels in Cre + mice suggests an impaired microglial response to amyloid pathology in the absence of astrocytic apoE.

### Astrocyte activation after the loss of astrocytic apoE

ApoE has been shown to influence certain functions of astrocytes, including responses to pathogenic stimuli [42, 43], however it is not fully known if the cell-specific loss of astrocytic apoE alters astrocyte reactivity to Aβ plaques. Therefore, we assessed the expression profile of genes involved with astrocyte reactivity by RT-qPCR. Overall expression of reactive astrocytic genes showed few differences between the Cre- and Cre + mice, including S100β (Fig. 5A). However, slight decreases of ~ 40–50% in GFAP expression were seen in the APPPS1;FE4Cre + mice, but not in the APPPS1;FE3Cre + mice (Fig. 5B). To visualize and further assess astrocyte reactivity, we assessed GFAP levels by immunostaining (Fig. 5C). Overall GFAP staining was not altered in APPPS1;FE3Cre + compared to APPPS1;FE3Cre- mice. However, there was a significant reduction in GFAP staining in the cortex of female APPPS1;FE4Cre + mice by 36% compared to APPPS1;FE4Cre- mice (Fig. 5B and C). The reductions in GFAP gene expression, and in immunostaining, in APPPS1;FE4Cre + mice could result from altered astrocyte reactivity to amyloid plaques or from overall reductions in amyloid burden. Therefore, to see if astrocyte reactivity was altered specifically around Aβ plaques, we assessed GFAP staining within 15 μm of each plaque (Fig. 5C). No differences were found in the levels of GFAP staining around plaques between the groups (Fig. 5E). Since GFAP staining around plaques was unaltered, it suggests the loss of apoE in astrocytes does not alter the ability of astrocytes to respond to Aβ plaques, at least morphologically, but that overall changes to astrocyte reactivity in the APPPS1;FE4Cre + mice may be driven by the level of total Aβ plaque pathology.

### Neuritic dystrophy after the loss of astrocytic apoE

The deposition of Aβ into fibrillar amyloid plaques results in damage to surrounding neuronal processes and leads to the formation of large swollen axons and dendrites around plaques (neuritic dystrophy). These damaged and swollen neurites contain accumulations of various proteins that can act as markers for the dystrophy, including BACE1 [44, 45] and RTN-3 [46, 47]. We used an anti-BACE1 antibody to stain and assess the total amount of dystrophic neurites present in the cortex of mice (Fig. 6A). The BACE1 levels for the female Cre + mice were significantly decreased by ~ 50–70% compared to the female Cre- mice (Fig. 6B). As the BACE-1 positive dystrophic neurites are only present around amyloid plaques, this is consistent with overall reductions in amyloid plaque following removal of astrocyte apoE. When we assessed the area of BACE1 within 15 μm of each plaque, we found a significant increase in neuritic dystrophy in the male Cre + mice of ~ 30% and an increase of 50% in female APPPS1;FE4Cre + mice (Fig. 6C and D). To further assess neuritic dystrophy around plaques, staining for RTN-3 was also performed (Fig. 6E, S5). The RTN-3 levels in female Cre + mice were found to be ~ 90–115% higher around plaques than in Cre- females (Fig. 6F).

## Discussion

The influence of *APOE* genotype on the development of various neurodegenerative pathologies has been an area of interest for decades. In particular, the strong effect that *APOE4* has on an individual’s risk for developing Alzheimer disease has been the focus of a tremendous amount of research. Clinical studies have shown that *APOE4* carriers develop an earlier onset and faster rate of development of Aβ pathology [17, 48]. Previous research has revealed several ways apoE may influence the buildup of Aβ and the formation of amyloid plaques [49, 50]. One way apoE can influence monomeric Aβ levels and its ultimate aggregation is by competing for clearance via apoE receptors, like the low-density lipoprotein receptor (LDLR) or LDL receptor-related protein 1 (LRP1) [12, 37, 51]. The ability of apoE to inhibit Aβ binding and clearance via LDLR and LRP1 is likely an important factor regulating the levels of Aβ in the brain. In addition, apoE can directly influence Aβ seeding and fibrillogenesis (i.e. the conversion of monomeric Aβ into oligomeric and fibrillar structures) [52–57]. However, it is not known if astrocytic apoE or microglial apoE may be having different effects on monomeric Aβ clearance and Aβ seeding/fibrillogenesis. While astrocytes are the primary producer of apoE in the CNS, microglia increase their production of apoE during the development of neurodegenerative pathology [28, 29]. Microglia produce and secrete species of human apoE that have altered glycosylation compared to astrocytes [58]. Additionally, the recent finding by our lab that microglia secrete human apoE-containing lipoprotein particles that are smaller and less lipidated than particles produced by astrocytes [33] points to the potential of apoE produced by each cell type having unique effects on the development of Aβ pathology.

For this study, we aimed to answer the question of how apoE3 and apoE4 specifically produced by astrocytes influence the formation of Aβ plaques and the subsequent response of cells to the amyloid pathology. In order to see how astrocytic apoE influences the initial stages of Aβ plaque development, we aimed to remove *APOE* prior to plaque onset. To ensure tamoxifen administration was occurring prior to the formation of Aβ plaque deposition, a cohort of tamoxifen injected mice was collected at 6 weeks of age, and Aβ immunostaining and X-34 staining showed no signs of any Aβ pathology. The 100 mg/kg dose of tamoxifen proved to be well tolerated and to efficiently lower *APOE* mRNA transcripts and to specifically lower most detectable protein levels of apoE from astrocytes in the Cre + mice. However, because insoluble apoE in the guanidine fraction is likely apoE bound to amyloid plaques [40, 41], the decrease in insoluble apoE in the guanidine fraction of the Cre + mice is likely due to overall Aβ plaque levels being decreased. While the tamoxifen administration did lower apoE levels overall, there was some variability seen in the efficiency of the injections. Immunostaining for apoE showed very efficient lowering of apoE and an absence of apoE in the majority of GFAP+ astrocytes; however, some mice had higher levels of apoE still present and some GFAP+ astrocytes also still stained positively for apoE. ApoE staining in the Cre- mice also appeared to show hippocampal levels of apoE to be close to cortical levels. While the pattern we observed of cortical plaque development at 6 weeks of age and hippocampal development at 3–4 months of age has been previously described [36], the large differences observed in plaque development despite the level of apoE appearing to be similar in both regions suggests other factors besides apoE may be important for regulating plaque deposition in the hippocampus. Nevertheless, the tamoxifen injections did prove effective in removing most astrocytic *APOE*, significantly lowering apoE levels, with a resultant strong decrease in Aβ pathology.

The reduction in Aβ plaque load observed by selectively decreasing astrocyte apoE3 and apoE4 levels is similar to the results seen in previous studies that had reduced apoE in a non-cell specific manner prior to plaque onset [15, 26]. However, it is worth noting that sex and apoE isoform-dependent differences were also observed. In particular, the apoE3 males had far lower insoluble Aβ levels and Aβ plaque load than the female and apoE4-expressing mice, which has been previously observed in both mice and humans [12, 59–62]. Interesting sex differences were also observed with the Cre- female mice having a greater amount of both apoE and Aβ_42_ in the guanidine fraction than the Cre- male mice, suggesting female mice may be more prone to producing apoE that accumulates with insoluble Aβ_42_. Further investigations into how sex might influence the role of astrocytic apoE plays in the development of Aβ pathology are certainly warranted. Additionally, we observed that the complete loss of apoE in APPPS1;EKO mice results in large deposits of diffuse non-fibrillar Aβ, similar to what we found previously [24], and that the level of diffuse Aβ deposition in the APPPS1;EKO compared to the selective removal of astrocyte apoE3 or apoE4 is considerably elevated. However, the very low X-34 to HJ3.4 ratio reveals that only a small fraction of the Aβ in APPPS1;EKO mice forms fibrillar plaques. The increased levels of non-fibrillar Aβ deposition in the APPPS1;EKO mice compared to APPPS1;FE3Cre + and APPPS1;FE4Cre + mice suggests that the presence of *APOE* in cells other than astrocytes may also be influencing overall Aβ deposition to some extent, though the strong decrease in overall Aβ accumulation resulting from the removal of astrocyte apoE3 and apoE4 suggests astrocytic apoE is a major driver of Aβ accumulation. While some data suggests that apoE can affect APP transcription and the production of Aβ [63], there was no change in the level of overall APP and APP CTF’s between any of the groups, indicating that the changes in Aβ plaque load with the loss of astrocytic apoE are likely not the result of changes in APP levels or in the production of Aβ. Furthermore, the decrease in the ratio of X-34 to HJ3.4 staining and the shift in fibrillar plaque formation towards less intense and less compact plaques with the loss of astrocytic *APOE* indicates astrocytic apoE is influencing the physical structure of the Aβ plaques that form. Since the structure of Aβ aggregates is likely driven by direct interactions of apoE and Aβ [52–55, 57], this suggests that a direct interaction between astrocyte apoE and some form of Aβ during the process of seeding or fibrillogenesis is involved in this Aβ structural change. While a complete loss of *APOE* has shown similar effects on fibrillar plaque levels and structure [14, 19, 24, 25], removing only astrocytic *APOE* in the APPPS1;FE3Cre + and APPPS1;FE4Cre + mice produced patterns of Aβ deposition distinct from the APPPS1;EKO. While very few β-sheet structured fibrils, as detected by X-34 [64], form in the APPPS1;EKO mice, those that do form, have a much more dispersed and mesh-like appearance than the fibrils that form in the Cre + mice. This suggests the presence or production of *APOE* in other cell types, such as microglia, may also be influencing the structure of Aβ plaque formation to some extent in Cre + mice. For example, fibrillar amyloid plaques formed in Cre + mice with strongly reduced astrocytic apoE still contain apoE, which indicates the apoE present in plaques is likely coming from other cellular sources such as microglia. In fact, a recent study showed that the removal of endogenous murine apoE from microglia results in the formation of dense-core plaques that are larger in size; however, there was no change in the overall levels of Aβ plaque load [65].

Another factor that could be impacting the fibrillar plaque structure is the ability of microglia to interact with the plaque surface. Previous studies have shown activated microglia are capable of ‘capping’ the edge of Aβ plaques to limit the diffusion of Aβ fibrils and aid in the formation of compact plaques [66, 67]. However, the low levels of microglial Clec7a and apoE around plaques in the Cre + mice suggests microglia are not activated to the same extent as in the Cre- mice. The shift in microglia activation to a reactive or DAM/MGnD state has been shown to be apoE dependent [28, 29, 68–70]. Furthermore, astrocytic apoE lipoprotein particles can influence microglial activation [71, 72], and our results support the hypothesis that astrocytic apoE may be involved in signaling pathways between astrocytes and microglia that regulate the apoE-dependent DAM activation state of microglia. The transition to the DAM activation state has been shown to be TREM2-APOE dependent [28] and apoE is a ligand for TREM2 [73–75]. Decreasing astrocytic apoE may subsequently lower overall TREM2 activation via decreased apoE binding. However, we did not observe a significant difference in the overall TREM2 transcript levels between the Cre- and Cre + groups and it is possible astrocyte-derived apoE may be involved in microglial activation via another yet to be determined mechanism. Additionally, since activated microglia are a source of plaque-associated apoE [34], decreased microglial activation may result in decreased microglial apoE production, as suggested by our results, and thus a reduction in the amount of microglial apoE binding to Aβ to aid in fibrillar plaque formation and plaque compaction.

The reduction in either astrocytic apoE3 or apoE4 had similar impacts on lowering Aβ plaque levels, as well as on reducing the activation of microglia surrounding Aβ plaques. This suggests that the influence of apoE isoforms on Aβ that have been previously observed in mouse models may not solely be driven by the expression of ApoE3 or ApoE4 by astrocytes, but rather by expression of apoE from other cell types, such as microglia. While the decrease in overall DAM gene expression in the Cre + mice may be driven by the overall Aβ plaque load being lower than the Cre- mice, the reduction in microglial activation around plaques, as marked by decreased Clec7a and apoE staining around plaques, indicates the loss of astrocytic apoE is having a local effect on the state of microglia as well. We also observed isoform- and sex-dependent differences in total astrocyte activation as measured by GFAP staining. Again, while the decrease in overall GFAP levels in the APPPS1;FE4Cre + mice may be explained by decreased Aβ plaque load, the lack of a difference in overall GFAP levels in APPPS1;FE3Cre + mice suggests that astrocytic apoE3 may be having a differential effect on reactive astrogliosis compared to astrocytic apoE4. Indeed, induced expression of apoE3 by astrocytes has been shown to lower overall GFAP levels [27]. It will be important in future studies to utilize methods such as single cell RNA sequencing of astrocytes, to more fully explore the apoE isoform and sex-dependent changes in reactive astrogliosis that are present in different disease conditions, including in the presence of amyloid [76].

The impact of apoE on microgliosis at sites of Aβ plaque deposition can lead to changes in damage to surrounding cells, including the formation of dystrophic neurites (neuritic dystrophy) [72]. Neuritic dystrophy has been shown to be increased around fibrillar plaques with a more dispersed morphology than around dense core plaques with a compact morphology [24, 66, 77–79]. The removal of astrocyte apoE strongly reduced the amount of fibrillar plaques and plaque-associated neuritic dystrophy, as indicated by reduced BACE1 and RTN-3 accumulation. However, the change in fibrillar plaque formation to a more dispersed and less compact structure observed in the APPPS1;FE3Cre + and APPPS1;FE4Cre + mice may be driving the increased neuritic dystrophy around the remaining fibrillar plaques present in these mice as is seen in the absence of apoE and TREM2 [24, 66]. Additionally, apoE lipoprotein particles produced by astrocytes have been shown to support neuronal function and recovery following neuronal damage [80–84]. The loss of astrocytic apoE could reduce astrocytic support to damaged neuritic processes that develop at sites of Aβ plaques and contribute to the increase in neuritic dystrophy. Finally, while Aβ plaques were detected in the hippocampus, and intense BACE1 staining of the mossy fibers of the hippocampus was observed, analysis of BACE1 in the hippocampus was not performed because of the small number of plaques present. The intense BACE1 mossy fiber staining has been previously reported and plays a role in normal physiological function and is not indicative of any neuronal damage akin to neuritic dystrophy [85–87]. However, given the decrease in overall neuritic dystrophy while dystrophy around plaques is increased, further studies are needed to better understand how the loss of astrocytic apoE is impacting overall neuronal function.

## Conclusion

We demonstrate that reducing astrocytic apoE3 and apoE4 results in a strong decrease in both fibrillar amyloid plaques and overall Aβ plaque deposition in APPPS1;FE3Cre + and APPPS1;FE4Cre + mice. We also found that the loss of astrocytic apoE results in the structure and pattern of Aβ deposition being altered in a unique manner compared to the complete loss of apoE in APPPS1;EKO mice. Furthermore, the decrease in microglial activation surrounding fibrillar plaques following removal of astrocyte apoE demonstrates astrocytic apoE may be playing a critical role in regulating microglial responses to Aβ pathology. Isoform-dependent effects on astrocyte activation were also seen and suggest astrocytic apoE3 may act to reduce overall astrocyte activation. Finally, while the more dispersed fibrillar plaque structures seen in the Cre + mice were shown to induce an increase in neuritic dystrophy at the site of Aβ plaque deposition, the ability of reducing astrocytic apoE to lower Aβ plaque levels led to an overall decrease in levels of neuritic dystrophy. These results demonstrate the therapeutic potential of using targeted cell-specific reduction of astrocytic apoE to ameliorate Aβ pathology that is found in Alzheimer disease.

## Supplementary Information

**Additional file 1 **: **Figure S1.** Fibrillar amyloid plaque staining in the cortex and hippocampus of male Cre- and Cre + mice. Representative images are of X-34 (blue) stained male brain sections. Scale bars = 1000 μm.

**Additional file 2 **: **Figure S2.** (**A**) Aβ plaque staining in the cortex and hippocampus of male Cre- and Cre + mice. Representative images are of Aβ immunostained male brain sections using the HJ3.4 anti-Aβ antibody (orange). Scale bars = 1000 μm. (**B**) Deposition pattern of Aβ plaque and fibrillar amyloid plaque staining in female Cre- and Cre + mice. Representative images are of X-34 (blue) and HJ3.4 (orange) co-stained brain sections. Scale bars = 50 μm. (**C**) Western blots of full-length APP and APP-CTFs for Cre- and Cre + mice. Images are of blots that used an anti-APP C-terminal antibody and an anti-α-tubulin antibody. For each group, *n* = 3 males and *n* = 3 females were used. (**D**) Level of APP and APP-CTFs in Cre- and Cre + mice. The density of each band was determined using ImageJ software. APP and APP-CTF values were normalized to α-tubulin (*n* = 6).

**Additional file 3 **: **Figure S3.** (**A-J**) Microglial gene expression analysis in Cre-, Cre + FE3Cre-, FE3Cre+, FE4Cre-, and FE4Cre + mice. Graphs are of genes assessed by qPCR from cortical tissue samples (*n* = 3–9). * *p* ≤ 0.05, ** *p* ≤ 0.01; three way ANOVA and uncorrected Fisher’s LSD test in (A-J). Data are expressed as mean ± SEM. See Supplementary Table 1 for detailed statistics.

**Additional file 4 **: **Figure S4.** Gene expression analysis of Serpina3n in Cre-, Cre + FE3Cre-, FE3Cre+, FE4Cre-, and FE4Cre + mice. Graph is of the Serpina3n gene assessed by qPCR from cortical tissue samples (*n* = 3–9). * *p* ≤ 0.05; three way ANOVA and uncorrected Fisher’s LSD test. Data are expressed as mean ± SEM. See Supplementary Table 1 for detailed statistics.

**Additional file 5 **: **Figure S5.** Dystrophic neurites around fibrillar amyloid plaques in Cre- and Cre + mice (RTN-3). Representative images are of male RTN-3 immunostaining (red), using an anti-RTN-3 antibody, around X-34 stained (blue) amyloid plaques. Scale bars = 20 μm.

**Additional file 6 **: **Supplementary Table 1**. Detailed statistic information.

## Data Availability

All data generated during this study have been included in the manuscript. Further data supporting the findings of this study are available from the corresponding authors on request.

## Abbreviations

AD: Alzheimer disease
Aβ: Amyloid beta
APOE: Apolipoprotein E
EKO: ApoE knock-out
GFAP: Glial fibrillary acidic protein
LDLR: Low-density lipoprotein receptor
LRP1: LDL receptor-related protein 1
DAM: Disease-associated microglia
ASO: Anti-sense oligonucleotides
BAC: Bacterial artificial chromosome
IBA1: ionized calcium binding adaptor molecule 1
SEM: Standard error of the mean
MGnD: Microglial neurodegenerative phenotype
TAM: Tamoxifen
PBS: Phosphate-buffered saline
TBS: Tris-buffered saline
TREM2: Triggering receptor expressed on myeloid cells 2
KO: Knock-out
ELISA: Enzyme-linked immunosorbent assay
ROI: Region of Interest
RTN-3: Reticulon-3
