# Supplementary figures and images for: Selective reduction of astrocyte apoE3 and apoE4 strongly reduces Aβ accumulation and plaque-related pathology in a mouse model of amyloidosis
Source: Mol Neurodegener. 2022 Feb 2;17:13. doi: 10.1186/s13024-022-00516-0 (PMC8811969; doi:10.1186/s13024-022-00516-0)

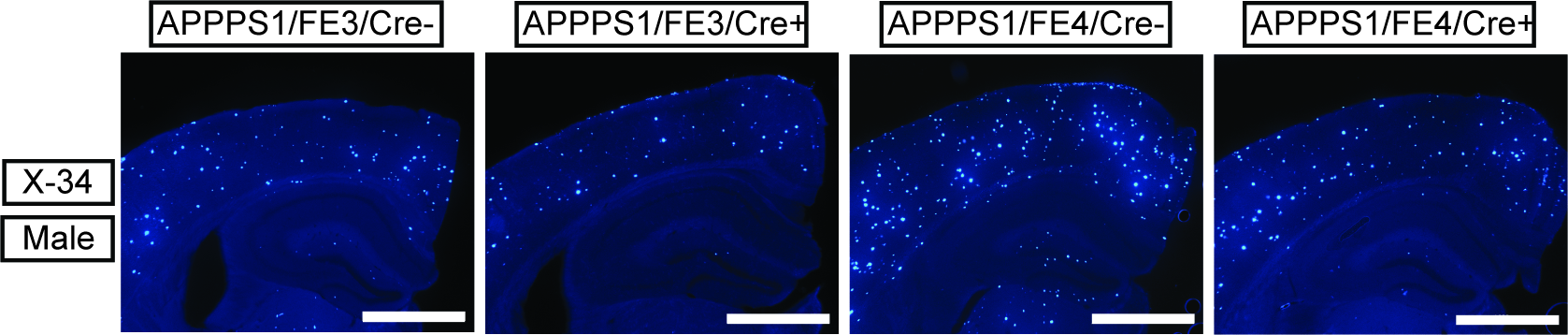

Supplement: Supplementary file 1 — Additional file 1 : Figure S1. Fibrillar amyloid plaque staining in the cortex and hippocampus of male Cre- and Cre + mice. Representative images are of X-34 (blue) stained male brain sections. Scale bars = 1000 μm. [file 13024_2022_516_MOESM1_ESM.tif]

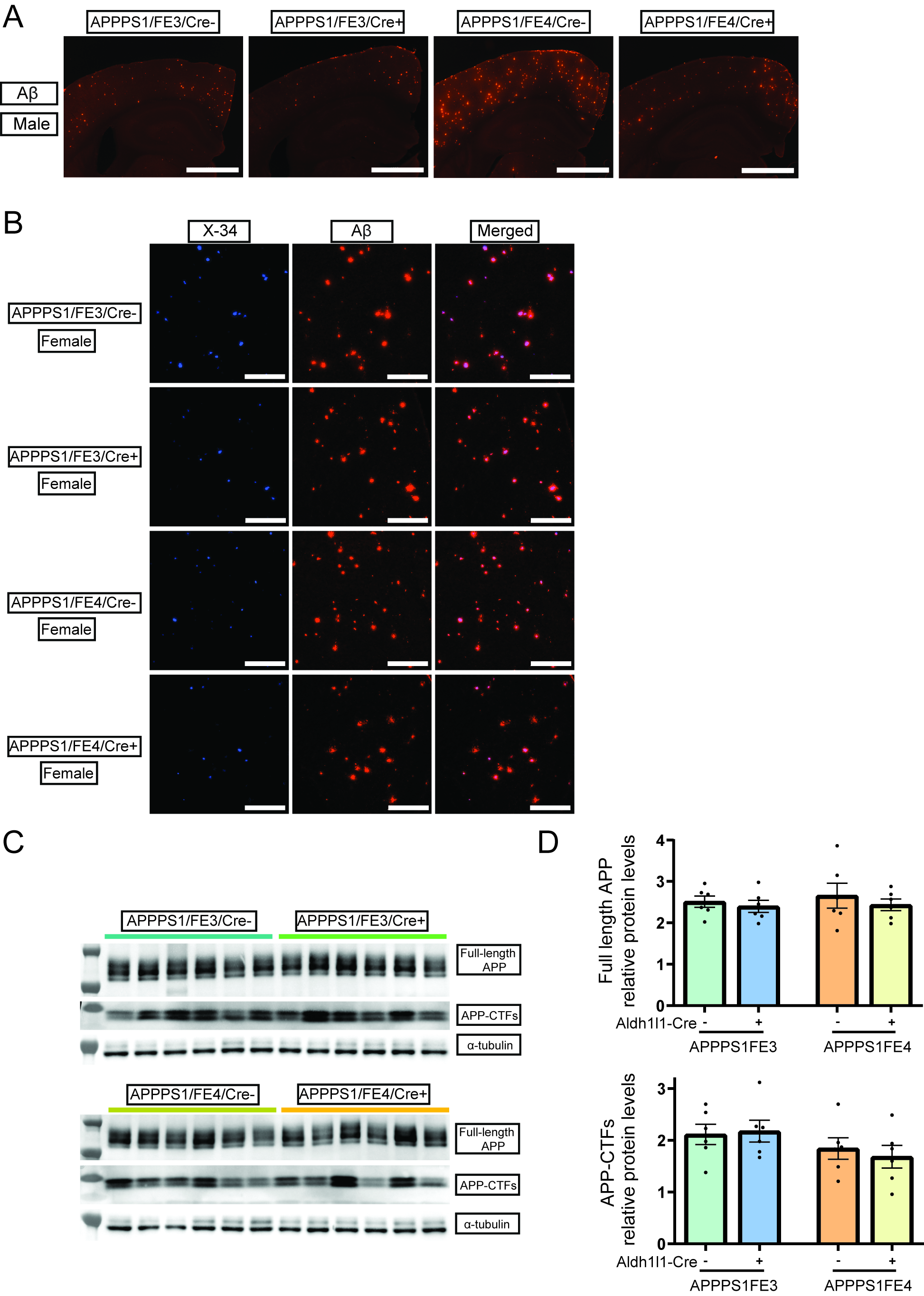

Supplement: Supplementary file 2 — Additional file 2 : Figure S2. (A) Aβ plaque staining in the cortex and hippocampus of male Cre- and Cre + mice. Representative images are of Aβ immunostained male brain sections using the HJ3.4 anti-Aβ antibody (orange). Scale bars = 1000 μm. (B) Deposition pattern of Aβ plaque and fibrillar amyloid plaque staining in female Cre- and Cre + mice. Representative images are of X-34 (blue) and HJ3.4 (orange) co-stained brain sections. Scale bars = 50 μm. (C) Western blots of full-length APP and APP-CTFs for Cre- and Cre + mice. Images are of blots that used an anti-APP C-terminal antibody and an anti-α-tubulin antibody. For each group, n = 3 males and n = 3 females were used. (D) Level of APP and APP-CTFs in Cre- and Cre + mice. The density of each band was determined using ImageJ software. APP and APP-CTF values were normalized to α-tubulin (n = 6). [file 13024_2022_516_MOESM2_ESM.tif]

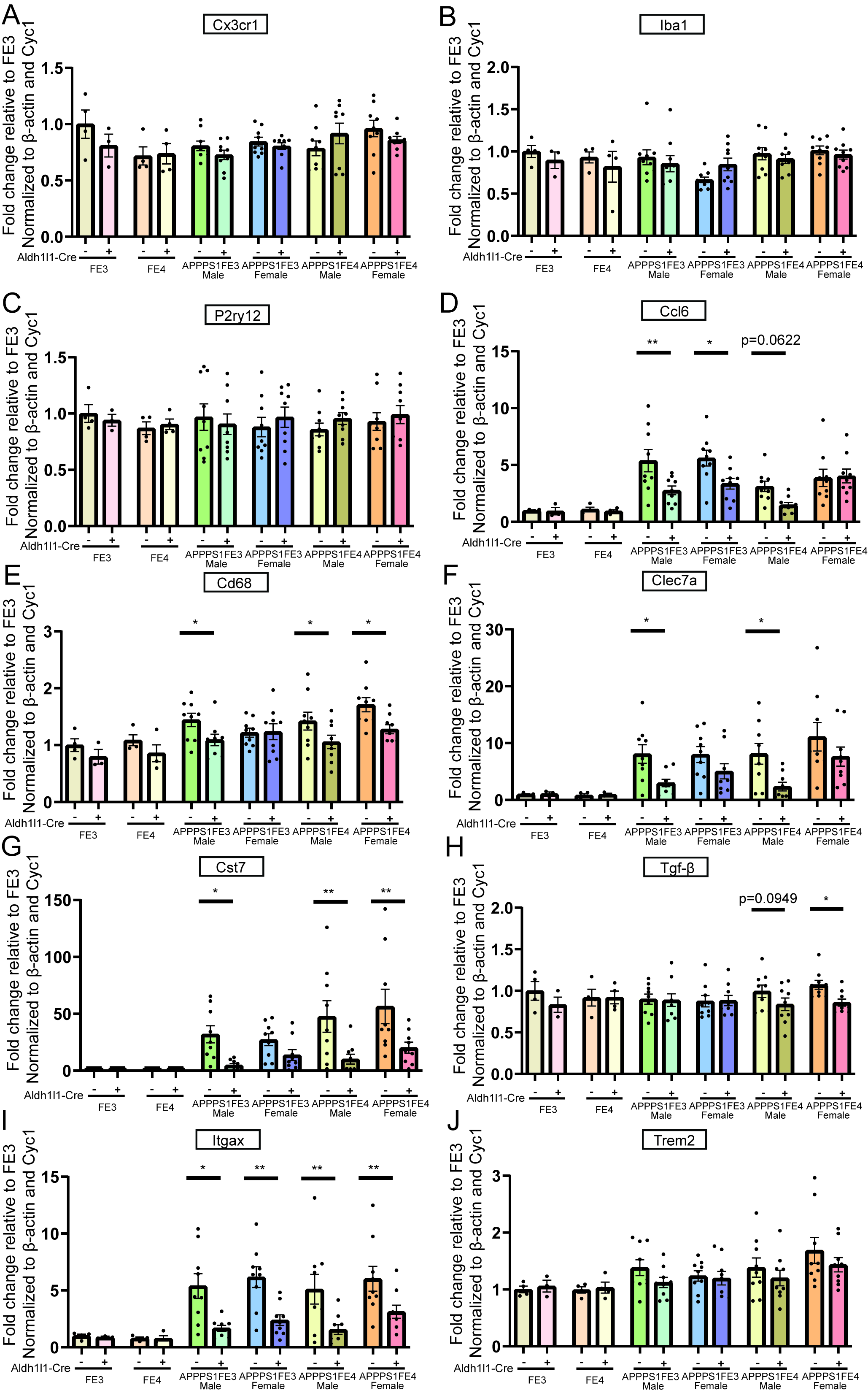

Supplement: Supplementary file 3 — Additional file 3 : Figure S3. (A-J) Microglial gene expression analysis in Cre-, Cre + FE3Cre-, FE3Cre+, FE4Cre-, and FE4Cre + mice. Graphs are of genes assessed by qPCR from cortical tissue samples (n = 3–9). * p ≤ 0.05, ** p ≤ 0.01; three way ANOVA and uncorrected Fisher’s LSD test in (A-J). Data are expressed as mean ± SEM. See Supplementary Table 1 for detailed statistics. [file 13024_2022_516_MOESM3_ESM.tif]

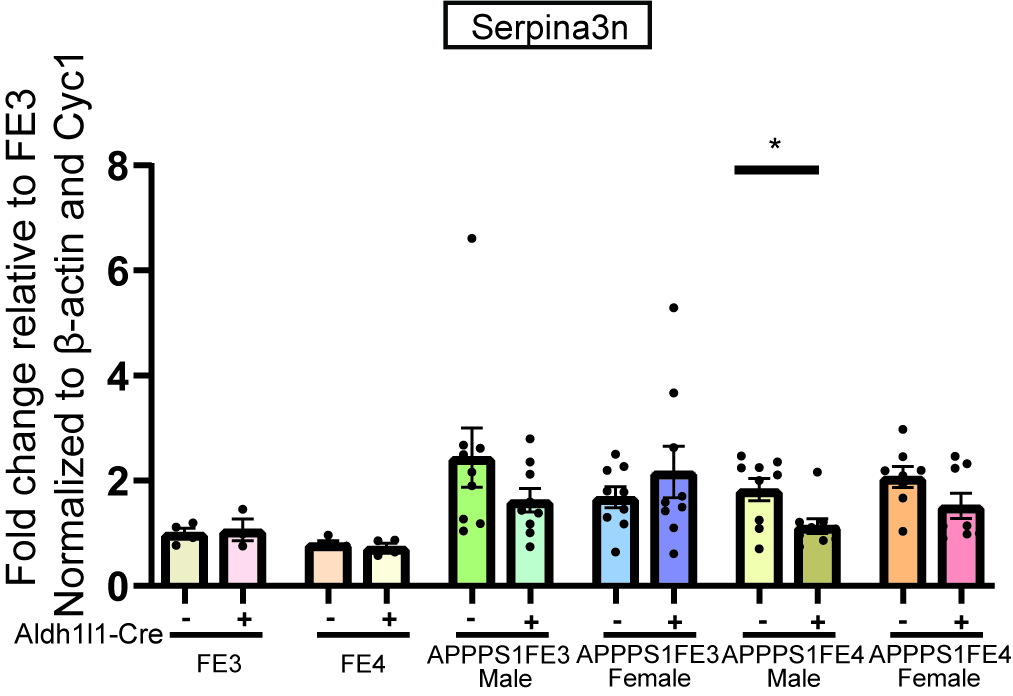

Supplement: Supplementary file 4 — Additional file 4 : Figure S4. Gene expression analysis of Serpina3n in Cre-, Cre + FE3Cre-, FE3Cre+, FE4Cre-, and FE4Cre + mice. Graph is of the Serpina3n gene assessed by qPCR from cortical tissue samples (n = 3–9). * p ≤ 0.05; three way ANOVA and uncorrected Fisher’s LSD test. Data are expressed as mean ± SEM. See Supplementary Table 1 for detailed statistics. [file 13024_2022_516_MOESM4_ESM.tif]

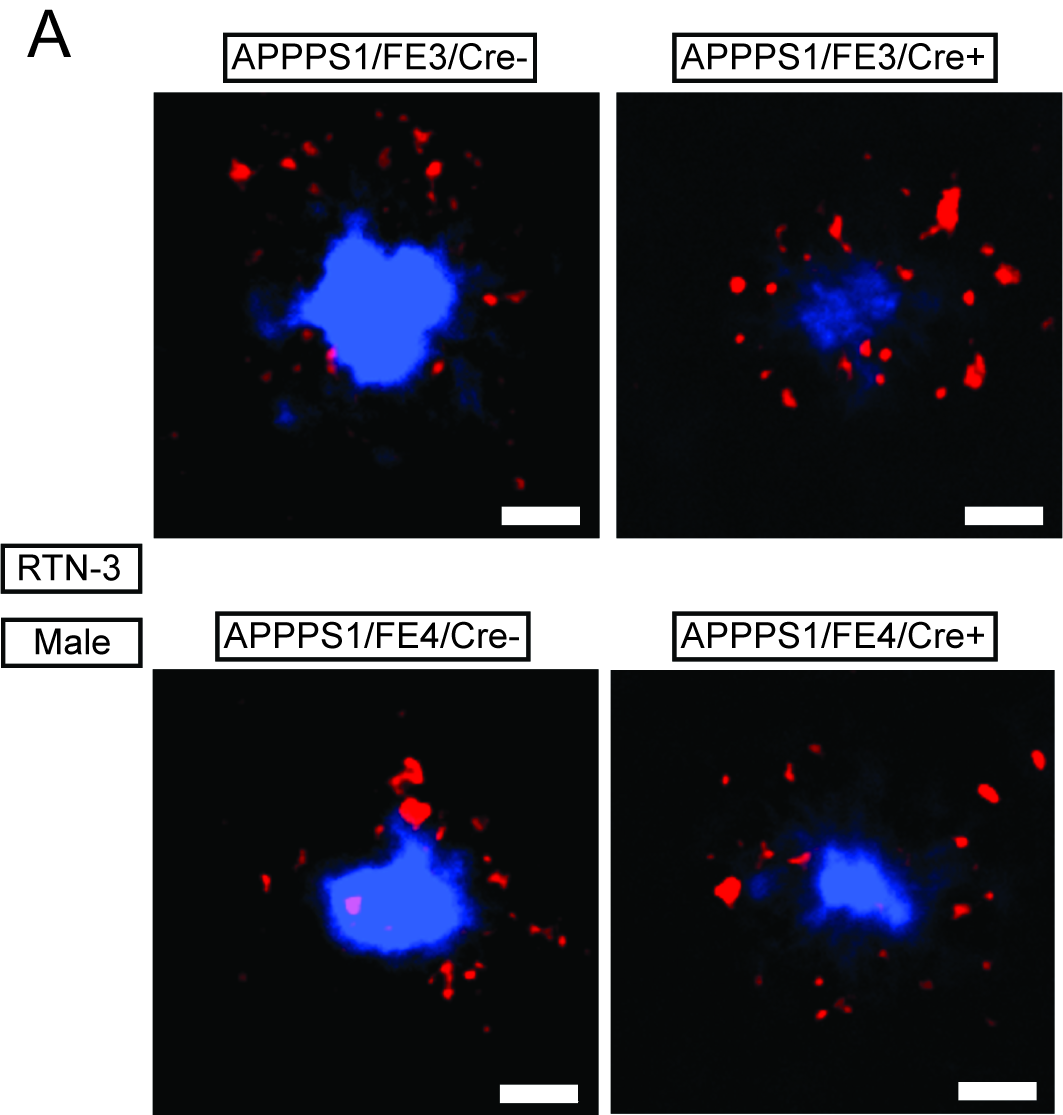

Supplement: Supplementary file 5 — Additional file 5 : Figure S5. Dystrophic neurites around fibrillar amyloid plaques in Cre- and Cre + mice (RTN-3). Representative images are of male RTN-3 immunostaining (red), using an anti-RTN-3 antibody, around X-34 stained (blue) amyloid plaques. Scale bars = 20 μm. [file 13024_2022_516_MOESM5_ESM.tif]
