# Supplementary material for: Selective reduction of astrocyte apoE3 and apoE4 strongly reduces Aβ accumulation and plaque-related pathology in a mouse model of amyloidosis
Source: Mol Neurodegener. 2022 Feb 2;17:13. doi: 10.1186/s13024-022-00516-0 (PMC8811969; doi:10.1186/s13024-022-00516-0)
Supplement: Supplementary file 6 — Additional file 6 : Supplementary Table 1. Detailed statistic information. [file 13024_2022_516_MOESM6_ESM.docx]

**Supplementary Table 1. Detailed statistic information.**

| Figure | Sample size | Statistic information table |
| --- | --- | --- |
| Figure 1B | N=9 per group | Cre F (1,64) = 252.8, p<0.0001; APOE F (1,64) = 4.944, p=0.0297; p<0.0001 in Male-APPPS1FE3Cre+ vs. Male-APPPS1FE3Cre-, Female-APPPS1FE3Cre+ vs. Female-APPPS1FE3Cre-, Male-APPPS1FE4Cre+ vs. Male-APPPS1FE4Cre-, Female-APPPS1FE4Cre+ vs. Female-APPPS1FE4Cre- |
| Figure 1C | Male-APPPS1FE4Cre-: n=10; Male-APPPS1FE4Cre+: n=14; Female-APPPS1FE4Cre-: n=14; Female-APPPS1FE4Cre+: n=9; Male-APPPS1FE3Cre-: n=19; Male-APPPS1FE3Cre+: n=10; Female-APPPS1FE3Cre-: n=10; Female-APPPS1FE3Cre+: n=10 | 1 sample from Male-APPPS1FE4Cre- and 1 sample from Female-APPPS1FE4Cre+ were excluded from final analysis according to the “identify outliers” function with Q=0.1%. Cre F (1,86) = 51.38, p<0.0001; APOE and Sex interaction F(1,86) = 5.511, p=0.0212; p=0.0010 in Male-APPPS1FE3Cre+ vs. Male-APPPS1FE3Cre-; p=0.0146 in Female-APPPS1FE3Cre+ vs. Female-APPPS1FE3Cre-; p=0.0001 in Male-APPPS1FE4Cre+ vs. Male-APPPS1FE4Cre-; p=0.0078 in Female-APPPS1FE4Cre+ vs. Female-APPPS1FE4Cre- |
| Figure 1D |  | Cre F (1,88) = 61.03, p<0.0001; Sex F(1,88) = 4.662, p=0.0336; Cre and Sex interaction F (1,88) = 6.714, p=0.0112; p=0.0332 in Male-APPPS1FE3Cre+ vs. Male-APPPS1FE3Cre-; p<0.0001 in Female-APPPS1FE3Cre+ vs. Female-APPPS1FE3Cre-; p=0.0281 in Male-APPPS1FE4Cre+ vs. Male-APPPS1FE4Cre-; p<0.0001 in Female-APPPS1FE4Cre+ vs. Female-APPPS1FE4Cre- |
| Figure 1F | Male-APPPS1FE4Cre-: n=10; Male-APPPS1FE4Cre+: n=14; Female-APPPS1FE4Cre-: n=14; Female-APPPS1FE4Cre+: n=10; Male-APPPS1FE3Cre-: n=19; Male-APPPS1FE3Cre+: n=10; Female-APPPS1FE3Cre-: n=10; Female-APPPS1FE3Cre+: n=10 | Cre F (1,89) = 77.81, p<0.0001; APOE F(1,89) = 5.272, p=0.0240; Cre and Sex interaction F(1,89) = 5.577, p=0.0204; p<0.0001 in Male-APPPS1FE3Cre+ vs. Male-APPPS1FE3Cre-, in Female-APPPS1FE3Cre+ vs. Female-APPPS1FE3Cre-, and in Female-APPPS1FE4Cre+ vs. Female-APPPS1FE4Cre-; p=0.0417 in Male-APPPS1FE4Cre+ vs. Male-APPPS1FE4Cre- |
| Figure 2B | Male-APPPS1FE4Cre-: n=10; Male-APPPS1FE4Cre+: n=14; Female-APPPS1FE4Cre-: n=11; Female-APPPS1FE4Cre+: n=10; Male-APPPS1FE3Cre-: n=18; Male-APPPS1FE3Cre+: n=10; Female-APPPS1FE3Cre-: n=10; Female-APPPS1FE3Cre+: n=10; APPPS1EKO: n=10 | Cre F (1,85) = 28.61, p<0.0001; Sex F(1,85) = 7.305, p=0.0083; p=0.0063 in Female-APPPS1FE3Cre+ vs. Female-APPPS1FE3Cre-; p=0.0012 in Male-APPPS1FE4Cre+ vs. Male-APPPS1FE4Cre-; p=0.0015 in Female-APPPS1FE4Cre+ vs. Female-APPPS1FE4Cre- |
| Figure 2C |  | Cre F (1,85) = 20.80, p<0.0001; Sex F(1,85) = 4.152, p=0.0447; p=0.0051 in Female-APPPS1FE3Cre+ vs. Female-APPPS1FE3Cre-; p=0.0419 in Male-APPPS1FE4Cre+ vs. Male-APPPS1FE4Cre-; p=0.0014 in Female-APPPS1FE4Cre+ vs. Female-APPPS1FE4Cre- |
| Figure 2E | Male-APPPS1FE4Cre-: n=10; Male-APPPS1FE4Cre+: n=14; Female-APPPS1FE4Cre-: n=11; Female-APPPS1FE4Cre+: n=10; Male-APPPS1FE3Cre-: n=16; Male-APPPS1FE3Cre+: n=9; Female-APPPS1FE3Cre-: n=10; Female-APPPS1FE3Cre+: n=10; APPPS1EKO: n=10 | 2 sample from Male-APPPS1FE3Cre- and 1 sample from Male-APPPS1FE3Cre+ were excluded from final analysis according to the “identify outliers” function with Q=0.1%. Cre F (1,82) = 71.6, p<0.0001; Sex F(1,82) = 11.31, p=0.0012; p=0.0032 in Male-APPPS1FE3Cre+ vs. Male-APPPS1FE3Cre-; p=0.0002 in Female-APPPS1FE3Cre+ vs. Female-APPPS1FE3Cre-; p<0.0001 in Male-APPPS1FE4Cre+ vs. Male-APPPS1FE4Cre-; p<0.0001 in Female-APPPS1FE4Cre+ vs. Female-APPPS1FE4Cre- |
| Figure 3B | Male-APPPS1FE4Cre-: n=10; Male-APPPS1FE4Cre+: n=14; Female-APPPS1FE4Cre-: n=11; Female-APPPS1FE4Cre+: n=10; Male-APPPS1FE3Cre-: n=18; Male-APPPS1FE3Cre+: n=10; Female-APPPS1FE3Cre-: n=10; Female-APPPS1FE3Cre+: n=10; APPPS1EKO: n=10 | Cre F (1,85) = 16.23, p=0.0001; APOE F (1,85) = 7.091, p=0.0093; Sex F(1,85) = 4.396, p=0.0396; p=0.0735 in Female-APPPS1FE3Cre+ vs. Female-APPPS1FE3Cre-; p=0.0163 in Male-APPPS1FE4Cre+ vs. Male-APPPS1FE4Cre-; p=0.0017 in Female-APPPS1FE4Cre+ vs. Female-APPPS1FE4Cre- |
| Figure 3C | Male-APPPS1FE4Cre-: n=10; Male-APPPS1FE4Cre+: n=14; Female-APPPS1FE4Cre-: n=14; Female-APPPS1FE4Cre+: n=10; Male-APPPS1FE3Cre-: n=18; Male-APPPS1FE3Cre+: n=10; Female-APPPS1FE3Cre-: n=10; Female-APPPS1FE3Cre+: n=10; APPPS1EKO: n=10 | Cre F (1,88) = 23.96, p<0.0001; APOE F (1,88) = 5.427, p=0.0221; Sex F(1,88) = 8.147, p=0.0054; Cre and Sex interaction F(1,88) = 6.426, p=0.0130; p=0.0151 in Female-APPPS1FE3Cre+ vs. Female-APPPS1FE3Cre-; p=0.0904 in Male-APPPS1FE4Cre+ vs. Male-APPPS1FE4Cre-; p<0.0001 in Female-APPPS1FE4Cre+ vs. Female-APPPS1FE4Cre- |
| Figure 3D | Male-APPPS1FE4Cre-: n=10; Male-APPPS1FE4Cre+: n=14; Female-APPPS1FE4Cre-: n=14; Female-APPPS1FE4Cre+: n=9; Male-APPPS1FE3Cre-: n=19; Male-APPPS1FE3Cre+: n=10; Female-APPPS1FE3Cre-: n=10; Female-APPPS1FE3Cre+: n=10 | Cre F (1,88) = 21.33, p<0.0001; APOE F (1,88) = 4.503, p=0.0366; Cre and Sex interaction F(1,88) = 4.098, p=0.0460; p=0.0403 in Female-APPPS1FE3Cre+ vs. Female-APPPS1FE3Cre-; p=0.0353 in Male-APPPS1FE4Cre+ vs. Male-APPPS1FE4Cre-; p<0.0001 in Female-APPPS1FE4Cre+ vs. Female-APPPS1FE4Cre- |
| Figure 3E |  | Cre F (1,88) = 13.31, p=0.0004; Sex F (1,88) = 4.439, p=0.0380; p=0.0048 in Female-APPPS1FE3Cre+ vs. Female-APPPS1FE3Cre-; p=0.0197 in Female-APPPS1FE4Cre+ vs. Female-APPPS1FE4Cre- |
| Figure 3G | Male-APPPS1FE4Cre-: n=10; Male-APPPS1FE4Cre+: n=10; Female-APPPS1FE4Cre-: n=14; Female-APPPS1FE4Cre+: n=10; Male-APPPS1FE3Cre-: n=16; Male-APPPS1FE3Cre+: n=6; Female-APPPS1FE3Cre-: n=10; Female-APPPS1FE3Cre+: n=10; APPPS1EKO: n=10 | Cre F (1,78) = 34.97, p<0.0001; Cre and Sex interaction F(1,78) = 5.476, p=0.0218; p=0.0002 in Male-APPPS1FE3Cre+ vs. Male-APPPS1FE3Cre-; p=0.0001 in Male-APPPS1FE4Cre+ vs. Male-APPPS1FE4Cre-; p=0.02 in Female-APPPS1FE4Cre+ vs. Female-APPPS1FE4Cre- |
| Figure 4B | Male-APPPS1FE4Cre-: n=10; Male-APPPS1FE4Cre+: n=14; Female-APPPS1FE4Cre-: n=14; Female-APPPS1FE4Cre+: n=10; Male-APPPS1FE3Cre-: n=19; Male-APPPS1FE3Cre+: n=10; Female-APPPS1FE3Cre-: n=10; Female-APPPS1FE3Cre+: n=10 | APOE F (1,89) = 10.94, p=0.0014; Sex F (1,89) = 84.79, p<0.0001; APOE and Sex interaction F(1,89) = 12.05, p=0.0008 |
| Figure 4D | Male-APPPS1FE4Cre-: n=8;  Male-APPPS1FE4Cre+: n=9; Female-APPPS1FE4Cre-: n=12; Female-APPPS1FE4Cre+: n=11; Male-APPPS1FE3Cre-: n=12; Male-APPPS1FE3Cre+: n=5; Female-APPPS1FE3Cre-: n=9; Female-APPPS1FE3Cre+: n=9 | 1 sample from Female-APPPS1FE4Cre+ and 1 sample from Male-APPPS1FE3Cre- were excluded from final analysis according to the “identify outliers” function with Q=0.1%. Cre F (1,65) = 39.7, p<0.0001; APOE F(1,65) = 5.227, p=0.0255; p=0.0025 in Male-APPPS1FE3Cre+ vs. Male-APPPS1FE3Cre-; p=0.0011 in Female-APPPS1FE3Cre+ vs. Female-APPPS1FE3Cre-; p=0.0129 in Male-APPPS1FE4Cre+ vs. Male-APPPS1FE4Cre-; p=0.0006 in Female-APPPS1FE4Cre+ vs. Female-APPPS1FE4Cre- |
| Figure 4F | Male-APPPS1FE4Cre-: n=9;  Male-APPPS1FE4Cre+: n=8; Female-APPPS1FE4Cre-: n=14; Female-APPPS1FE4Cre+: n=8; Male-APPPS1FE3Cre-: n=12; Male-APPPS1FE3Cre+: n=6; Female-APPPS1FE3Cre-: n=9; Female-APPPS1FE3Cre+: n=8 | Sex F(1,66) = 4.146, p=0.0457 |
| Figure 4G |  | Cre F (1,66) = 25.21, p<0.0001; APOE F (1,66) = 4.132, p=0.0461; p=0.0580 in Male-APPPS1FE3Cre+ vs. Male-APPPS1FE3Cre-; p=0.0189 in Female-APPPS1FE3Cre+ vs. Female-APPPS1FE3Cre-; p=0.0005 in Male-APPPS1FE4Cre+ vs. Male-APPPS1FE4Cre-; p=0.0500 in Female-APPPS1FE4Cre+ vs. Female-APPPS1FE4Cre- |
| Figure 5A | N=4 in APPPS1 negative, FE3Cre-, FE4Cre-, and FE4Cre+ groups;  N=3 in APPPS1 negative FE3Cre+;  N=9 in all APPPS1 positive groups | N/A |
| Figure 5B |  | 1 sample from Female-APPPS1FE4Cre+ was excluded from final analysis according to the “identify outliers” function with Q=0.1%. Cre F (1,63) = 4.396, p=0.0401; APOE F(1,63) = 4.006, p=0.0497; Sex F(1,63) = 4.487, p=0.0381; p=0.0338 in Male-APPPS1FE4Cre+ vs. Male-APPPS1FE4Cre-; p=0.0694 in Female-APPPS1FE4Cre+ vs. Female-APPPS1FE4Cre- |
| Figure 5D | Male-APPPS1FE4Cre-: n=10; Male-APPPS1FE4Cre+: n=14; Female-APPPS1FE4Cre-: n=14; Female-APPPS1FE4Cre+: n=10; Male-APPPS1FE3Cre-: n=19; Male-APPPS1FE3Cre+: n=10; Female-APPPS1FE3Cre-: n=10; Female-APPPS1FE3Cre+: n=10 | Cre F (1,89) = 3.302, p=0.0725; APOE F(1,89) = 15.68, p=0.0002; Sex F(1,89) = 5.729, p=0.0188; Cre, APOE, and Sex interaction F(1,89) = 3.994, p=0.0487; p=0.0077 in Female-APPPS1FE4Cre+ vs. Female-APPPS1FE4Cre- |
| Figure 5E | Male-APPPS1FE4Cre-: n=11; Male-APPPS1FE4Cre+: n=10; Female-APPPS1FE4Cre-: n=14; Female-APPPS1FE4Cre+: n=10; Male-APPPS1FE3Cre-: n=19; Male-APPPS1FE3Cre+: n=8; Female-APPPS1FE3Cre-: n=8; Female-APPPS1FE3Cre+: n=9 | N/A |
| Figure 6B | Male-APPPS1FE4Cre-: n=10; Male-APPPS1FE4Cre+: n=14; Female-APPPS1FE4Cre-: n=14; Female-APPPS1FE4Cre+: n=10; Male-APPPS1FE3Cre-: n=19; Male-APPPS1FE3Cre+: n=10; Female-APPPS1FE3Cre-: n=10; Female-APPPS1FE3Cre+: n=10 | Cre F (1,89) = 17.93, p<0.0001; Sex F (1,89) = 8.648, p=0.0042; p=0.0051 in Female-APPPS1FE3Cre+ vs. Female-APPPS1FE3Cre-; p=0.0121 in Female-APPPS1FE4Cre+ vs. Female-APPPS1FE4Cre- |
| Figure 6D | Male-APPPS1FE4Cre-: n=10; Male-APPPS1FE4Cre+: n=10; Female-APPPS1FE4Cre-: n=14; Female-APPPS1FE4Cre+: n=10; Male-APPPS1FE3Cre-: n=18; Male-APPPS1FE3Cre+: n=6; Female-APPPS1FE3Cre-: n=10; Female-APPPS1FE3Cre+: n=9 | Cre F (1,79) = 18.20, p<0.0001; p=0.0461 in Male-APPPS1FE3Cre+ vs. Male-APPPS1FE3Cre-; p=0.0316 in Male-APPPS1FE4Cre+ vs. Male-APPPS1FE4Cre-; p=0.0003 in Female-APPPS1FE4Cre+ vs. Female-APPPS1FE4Cre- |
| Figure 6F | Male-APPPS1FE4Cre-: n=10; Male-APPPS1FE4Cre+: n=8; Female-APPPS1FE4Cre-: n=14; Female-APPPS1FE4Cre+: n=8; Male-APPPS1FE3Cre-: n=12; Male-APPPS1FE3Cre+: n=6; Female-APPPS1FE3Cre-: n=9; Female-APPPS1FE3Cre+: n=8 | Cre F (1,67) = 9.448, p=0.0031; Cre and Sex interaction F(1,67) = 6.594, p=0.0125; p=0.0002 in Male-APPPS1FE3Cre+ vs. Male-APPPS1FE3Cre-; p=0.0058 in Female-APPPS1FE3Cre+ vs. Female-APPPS1FE3Cre-; p=0.0045 in Female-APPPS1FE4Cre+ vs. Female-APPPS1FE4Cre- |
